# Supplementary material for: Early life stress shifts critical periods and causes precocious visual cortex development
Source: PLoS One. 2024 Dec 31;19(12):e0316384. doi: 10.1371/journal.pone.0316384 (PMC11687811; doi:10.1371/journal.pone.0316384)
Supplement: S1 Table — Included are FDR-adjusted values with p<0.1 in order of smallest adjusted p-values. ‡ Indicates mRNA transcripts that were differentially expressed in more than one time-point. (DOCX) [file pone.0316384.s001.docx]

**Supplemental Table 1.** mRNA expression fold change (log_2_) with raw and adjusted *p*-values. Included are FDR-adjusted values with *p* < 0.1 in order of smallest adjusted *p*-values. ‡Indicates mRNA transcripts that were differentially expressed in more than one time-point.

| **Age** | **mRNA** | **Fold Change (log_2_)** | **Standard Error** | **Wald Statistic** | ***p*** | **Adjusted *p*** |
| --- | --- | --- | --- | --- | --- | --- |
| P20 | Dusp1 | -2.513 | 0.578 | 18.646 | 8.933E-05 | 9.907E-02 |
| P20 | Gem | -2.222 | 0.825 | 18.348 | 1.037E-04 | 9.907E-02 |
| P20 | Garem1 | -0.755 | 0.171 | 19.978 | 4.590E-05 | 9.907E-02 |
| P20 | Csnk1g1 | -0.519 | 0.130 | 19.158 | 6.918E-05 | 9.907E-02 |
| P20 | Tef‡ | 0.352 | 0.087 | 18.708 | 8.664E-05 | 9.907E-02 |
| P20 | Gas6 | 0.356 | 0.084 | 18.334 | 1.044E-04 | 9.907E-02 |
| P20 | Mgat5b‡ | 0.383 | 0.121 | 24.716 | 4.295E-06 | 2.648E-02 |
| P20 | Plbd2 | 0.414 | 0.097 | 18.504 | 9.593E-05 | 9.907E-02 |
| P20 | Lin7b | 0.533 | 0.124 | 19.544 | 5.703E-05 | 9.907E-02 |
| P20 | Neurl1b | 0.703 | 0.152 | 23.259 | 8.900E-06 | 3.659E-02 |
| P20 | Hrh3‡ | 0.737 | 0.171 | 19.070 | 7.229E-05 | 9.907E-02 |
| P20 | Deaf1 | 0.757 | 0.142 | 28.233 | 7.401E-07 | 9.127E-03 |
| P20 | Col8a1 | 1.545 | 0.354 | 19.850 | 4.893E-05 | 9.907E-02 |
| P35 | Hbb-bs | -1.261 | 0.331 | -3.807 | 1.407E-04 | 9.612E-02 |
| P35 | Eln | -0.616 | 0.156 | -3.940 | 8.165E-05 | 7.440E-02 |
| P35 | Sstr3 | -0.347 | 0.090 | -3.863 | 1.121E-04 | 8.247E-02 |
| P35 | Vhl | -0.257 | 0.068 | -3.774 | 1.605E-04 | 9.994E-02 |
| P35 | Faim2 | 0.204 | 0.043 | 4.701 | 2.590E-06 | 6.191E-03 |
| P35 | Ints3 | 0.213 | 0.048 | 4.388 | 1.145E-05 | 1.824E-02 |
| P35 | Atg9a | 0.251 | 0.065 | 3.879 | 1.047E-04 | 8.247E-02 |
| P35 | Mta3 | 0.257 | 0.053 | 4.821 | 1.431E-06 | 6.191E-03 |
| P35 | L1cam | 0.295 | 0.073 | 4.043 | 5.276E-05 | 6.305E-02 |
| P35 | Mgat5b‡ | 0.316 | 0.080 | 3.928 | 8.559E-05 | 7.440E-02 |
| P35 | Rhobtb2 | 0.332 | 0.069 | 4.822 | 1.423E-06 | 6.191E-03 |
| P35 | Hlf | 0.349 | 0.081 | 4.288 | 1.804E-05 | 2.464E-02 |
| P35 | Ahsa2 | 0.395 | 0.105 | 3.764 | 1.672E-04 | 9.994E-02 |
| P35 | St8sia1 | 0.407 | 0.103 | 3.943 | 8.042E-05 | 7.440E-02 |
| P35 | Cys1 | 0.535 | 0.113 | 4.724 | 2.315E-06 | 6.191E-03 |
| P35 | Lonrf1 | 0.600 | 0.137 | 4.389 | 1.137E-05 | 1.824E-02 |
| P50 | Cbln3 | -4.762 | 1.155 | -4.123 | 3.742E-05 | 9.917E-02 |
| P50 | Cyr61 | -3.023 | 0.643 | -4.700 | 2.599E-06 | 2.324E-02 |
| P50 | Gadd45g | -1.005 | 0.244 | -4.114 | 3.883E-05 | 9.917E-02 |
| P50 | Ppp1r3c | -0.545 | 0.121 | -4.489 | 7.170E-06 | 4.273E-02 |
| P50 | Tef‡ | 0.243 | 0.059 | 4.122 | 3.761E-05 | 9.917E-02 |
| P50 | B930095G15Rik | 0.416 | 0.096 | 4.348 | 1.375E-05 | 6.145E-02 |
| P50 | Hrh3‡ | 0.567 | 0.116 | 4.907 | 9.252E-07 | 1.654E-02 |
