# Supplementary material for: Early life stress shifts critical periods and causes precocious visual cortex development
Source: PLoS One. 2024 Dec 31;19(12):e0316384. doi: 10.1371/journal.pone.0316384 (PMC11687811; doi:10.1371/journal.pone.0316384)
Supplement: S2 Table — Metabolite regulation is shown as a function of relative concentration in high-EPS individuals. Metabolites for which more than one NMR resonance peak was identified as significant are represented as metabolite.1, metabolite.2, … metabolite.n. * Indicates metabolites that were significantly altered by stress in both left and right cerebra. (DOCX) [file pone.0316384.s002.docx]

**Supplemental Table 2.** Left and right cerebrum metabolites found to be significantly altered by stress in a Mann-Whitney U test. Metabolite regulation is shown as a function of relative concentration in high-EPS individuals. Metabolites for which more than one NMR resonance peak was identified as significant are represented as metabolite.1, metabolite.2, … metabolite.n. *Indicates metabolites that were significantly altered by stress in both left and right cerebra.

| **Region** | **Metabolite** | **NMR Chemical Shift Range of Bin (ppm)** | **Mann-Whitney U Test** | **Percent Difference** | **Regulation by Stress** |
| --- | --- | --- | --- | --- | --- |
| Left Cerebrum | Singlet 3.055 ppm | 3.060-3.049 | 8.25E-05 | -72.52 | Down |
|  | Adenosine.1* | 8.268-8.259 | 8.25E-05 | -55.10 | Down |
|  | Adenosine.2* | 6.119-6.108 | 8.25E-05 | -52.74 | Down |
|  | Adenosine.3* | 6.108-6.097 | 1.07E-04 | -51.29 | Down |
|  | Adenosine.4* | 8.378-8.354 | 8.25E-05 | -48.99 | Down |
|  | Glutamate.1* | 2.358-2.348 | 8.25E-05 | -44.31 | Down |
|  | Glutamate.2* | 2.348-2.336 | 8.25E-05 | -43.76 | Down |
|  | Adenosine.5* | 4.457-4.449 | 8.25E-05 | -43.51 | Down |
|  | Formate* | 8.475-8.453 | 8.25E-05 | -43.31 | Down |
|  | Adenosine.6* | 4.449-4.444 | 8.25E-05 | -39.61 | Down |
|  | Glutamate.3* | 2.336-2.328 | 8.25E-05 | -38.98 | Down |
|  | N-Acetylaspartate.1* | 7.992-7.952 | 8.25E-05 | -38.24 | Down |
|  | Adenosine.7* | 4.398-4.389 | 8.25E-05 | -38.13 | Down |
|  | Adenosine.8* | 4.389-4.384 | 8.25E-05 | -37.86 | Down |
|  | Adenosine.9* | 4.290-4.284 | 8.25E-05 | -37.30 | Down |
| Left  Cerebrum | Carnitine.1* | 4.463-4.457 | 8.25E-05 | -37.22 | Down |
|  | Adenosine.10* | 4.405-4.398 | 8.25E-05 | -37.05 | Down |
|  | Fumarate* | 6.535-6.518 | 5.97E-04 | -37.03 | Down |
|  | N-Acetylaspartate.2* | 2.681-2.677 | 8.25E-05 | -35.31 | Down |
|  | N-Acetylaspartate.3* | 2.687-2.681 | 8.25E-05 | -35.05 | Down |
|  | Glutamate.4*, N-Acetylaspartate.4*, Pyroglutamate.1* | 2.035-2.013 | 8.25E-05 | -34.45 | Down |
|  | N-Acetylaspartate.5* | 4.379-4.373 | 8.25E-05 | -34.15 | Down |
|  | Adenosine.11* | 4.410-4.405 | 8.25E-05 | -33.95 | Down |
|  | N-Acetylaspartate.6*, Pyroglutamate.2* | 2.514-2.507 | 3.72E-04 | -32.83 | Down |
|  | Adenosine.12* | 4.384-4.379 | 8.25E-05 | -30.41 | Down |
|  | Serine.1* | 3.996-3.989 | 8.25E-05 | -30.21 | Down |
|  | Glutamate.5* | 2.372-2.362 | 8.25E-05 | -29.68 | Down |
|  | Adenosine.13* | 4.284-4.274 | 8.25E-05 | -28.95 | Down |
|  | Glutamate.6*, Glycerol.1* | 3.772-3.766 | 8.25E-05 | -28.83 | Down |
|  | Pyroglutamate.3* | 2.533-2.521 | 4.72E-04 | -27.71 | Down |
|  | N-Acetylaspartate.7* | 2.768-2.759 | 8.25E-05 | -26.81 | Down |
|  | Adenosine.14* | 4.295-4.290 | 8.25E-05 | -26.44 | Down |
|  | Glutamate.7* | 2.085-2.075 | 8.25E-05 | -25.67 | Down |
|  | Glutamate.8* | 2.321-2.317 | 8.25E-05 | -24.83 | Down |
|  | Pyroglutamate.4* | 4.193-4.181 | 6.14E-03 | -24.76 | Down |
|  | Pyroglutamate.5* | 4.181-4.166 | 5.97E-04 | -24.59 | Down |
|  | Glutamate.9*, Glycerol.2* | 3.766-3.757 | 8.25E-05 | -24.50 | Down |
| Left Cerebrum | Myo-Inositol.1* | 3.370-3.360 | 1.07E-04 | -23.98 | Down |
|  | Serine.2* | 3.989-3.980 | 1.07E-04 | -23.03 | Down |
|  | Pyroglutamate.6* | 2.379-2.372 | 8.25E-05 | -22.88 | Down |
|  | Carnitine.2* | 3.232-3.217 | 1.18E-03 | -22.38 | Down |
|  | Creatine*, Tyrosine.1* | 3.931-3.919 | 8.25E-05 | -21.79 | Down |
|  | Pyroglutamate.7* | 4.166-4.162 | 3.72E-04 | -21.61 | Down |
|  | Glutamate.10* | 2.325-2.321 | 8.25E-05 | -21.53 | Down |
|  | Pyroglutamate.8* | 4.202-4.193 | 1.28E-02 | -21.36 | Down |
|  | N-Acetylaspartate.8* | 4.373-4.368 | 1.82E-03 | -20.35 | Down |
|  | O-Phosphocholine.1 | 4.162-4.159 | 2.92E-04 | -20.24 | Down |
|  | N-Acetylaspartate.9* | 4.368-4.359 | 9.42E-04 | -19.97 | Down |
|  | Glutamate.11* | 2.119-2.107 | 2.92E-04 | -19.70 | Down |
|  | N-Acetylaspartate.10*, Pyroglutamate.9* | 2.507-2.498 | 4.15E-03 | -18.66 | Down |
|  | Glutamate.12* | 2.094-2.085 | 8.25E-05 | -18.42 | Down |
|  | O-Phosphocholine.2 | 4.159-4.155 | 5.97E-04 | -18.03 | Down |
|  | Glutamate.13* | 2.064-2.056 | 8.25E-05 | -17.34 | Down |
|  | Carnitine.3*, Levulinate.1*, Pyroglutamate.10* | 2.402-2.391 | 2.15E-02 | -17.15 | Down |
|  | N-Acetylaspartate.11* | 4.355-4.347 | 6.14E-03 | -16.98 | Down |
|  | Adenosine.15* | 4.300-4.295 | 7.51E-04 | -16.56 | Down |
|  | Creatinine.1* | 4.051-4.044 | 9.42E-04 | -16.52 | Down |
|  | Glutamate.14* | 2.362-2.358 | 8.25E-05 | -16.47 | Down |
|  | Adenosine.16* | 4.304-4.300 | 9.42E-04 | -16.33 | Down |
| Left  Cerebrum | Lactate.1* | 4.114-4.106 | 1.07E-04 | -16.06 | Down |
|  | Adenosine.17* | 4.320-4.314 | 1.82E-03 | -15.98 | Down |
|  | Adenosine.18* | 4.314-4.309 | 1.82E-03 | -15.91 | Down |
|  | Serine.3* | 4.031-4.025 | 1.78E-04 | -15.60 | Down |
|  | Lactate.2* | 4.125-4.114 | 1.07E-04 | -15.37 | Down |
|  | N-Acetylaspartate.12* | 4.359-4.355 | 1.07E-02 | -14.88 | Down |
|  | O-Phosphocholine.3, Threonine.1* | 3.596-3.591 | 3.40E-03 | -14.88 | Down |
|  | Tyrosine.2* | 3.960-3.953 | 8.25E-05 | -14.21 | Down |
|  | Glutamate.15* | 2.075-2.064 | 8.25E-05 | -13.95 | Down |
|  | D-Threitol.1* | 3.723-3.719 | 7.51E-04 | -13.05 | Down |
|  | N-Acetylaspartate.13* | 2.711-2.697 | 1.78E-04 | -12.62 | Down |
|  | 4-Pyridoxate.1* | 7.898-7.890 | 4.06E-02 | -12.53 | Down |
|  | Serine.4* | 4.039-4.031 | 1.38E-04 | -12.53 | Down |
|  | Glutamate.16* | 2.129-2.119 | 3.40E-03 | -12.50 | Down |
|  | O-Phosphocholine.4 | 4.136-4.125 | 1.78E-04 | -12.22 | Down |
|  | Carnitine.4*, Levulinate.2*, Pyroglutamate.11* | 2.391-2.385 | 8.25E-05 | -12.13 | Down |
|  | Glutamate.17*, Pyroglutamate.12* | 2.056-2.045 | 1.82E-03 | -11.65 | Down |
|  | Glutamate.18* | 2.107-2.100 | 9.42E-04 | -10.81 | Down |
|  | Threonine.2* | 4.247-4.240 | 2.15E-02 | -10.77 | Down |
|  | N-Acetylaspartate.14* | 2.669-2.664 | 1.78E-04 | -10.02 | Down |
|  | Lactate.3* | 4.106-4.101 | 5.97E-04 | -9.64 | Down |
|  | Lactate.4*, Threonine.3* | 1.351-1.319 | 1.82E-03 | -9.56 | Down |
| Left  Cerebrum | Adenosine.19* | 8.278-8.268 | 5.06E-03 | -9.41 | Down |
|  | 4-Pyridoxate.2* | 7.887-7.878 | 3.48E-02 | -9.02 | Down |
|  | Adenosine.20* | 8.259-8.249 | 2.53E-02 | -8.96 | Down |
|  | N-Acetylaspartate.15* | 2.664-2.660 | 5.97E-04 | -8.52 | Down |
|  | Glycerol.3* | 3.647-3.638 | 1.47E-03 | -8.15 | Down |
|  | 1,3-Dimethylurate.1* | 3.241-3.232 | 4.15E-03 | -7.94 | Down |
|  | Myo-Inositol.2* | 4.093-4.055 | 1.82E-03 | -7.81 | Down |
|  | D-Threitol.2*, Glycerol.4*, Myo-Inositol.3* | 3.638-3.622 | 1.82E-03 | -7.33 | Down |
|  | D-Threitol.3* | 3.702-3.699 | 2.25E-03 | -7.27 | Down |
|  | Lactate.5* | 4.098-4.093 | 1.53E-02 | -7.18 | Down |
|  | Tyrosine.3* | 3.947-3.931 | 9.42E-04 | -6.99 | Down |
|  | Glycerol.5*, Myo-Inositol.4* | 3.550-3.538 | 2.77E-03 | -6.83 | Down |
|  | N-Acetylaspartate.16*, Pyroglutamate.13* | 2.498-2.485 | 1.53E-02 | -6.77 | Down |
|  | Myo-Inositol.5* | 3.428-3.417 | 1.53E-02 | -6.77 | Down |
|  | Myo-Inositol.6* | 3.289-3.278 | 8.94E-03 | -6.61 | Down |
|  | O-Phosphocholine.5 | 4.148-4.136 | 3.72E-04 | -6.57 | Down |
|  | Myo-Inositol.7* | 3.441-3.428 | 1.28E-02 | -6.52 | Down |
|  | 1,3-Dimethylurate.2*, Myo-Inositol.8* | 3.304-3.289 | 5.06E-03 | -6.14 | Down |
|  | D-Threitol.4*, Myo-Inositol.9* | 3.622-3.611 | 2.77E-03 | -5.69 | Down |
|  | Glycerol.6*, Glycine | 3.563-3.550 | 4.15E-03 | -4.89 | Down |
|  | Creatinine.2* | 3.049-3.027 | 5.06E-03 | -4.77 | Down |
|  | Carnitine.5*, Levulinate.3*, Pyroglutamate.14* | 2.385-2.379 | 2.98E-02 | -3.74 | Down |
| Left  Cerebrum | D-Threitol.5* | 3.719-3.712 | 2.53E-02 | -2.25 | Down |
|  | Homoserine.19* | 2.013-2.004 | 1.82E-02 | 4.51 | Up |
|  | 4-Aminobutyrate.10* | 2.306-2.294 | 6.14E-03 | 4.96 | Up |
|  | Isoleucine.13* | 3.677-3.670 | 2.25E-03 | 5.25 | Up |
|  | β-Alanine.6* | 2.571-2.565 | 7.42E-03 | 5.42 | Up |
|  | Aspartate.9*, Uridine.8* | 3.900-3.883 | 7.51E-04 | 5.55 | Up |
|  | 2-Oxoglutarate.5*, 4-Aminobutyrate.9* | 3.016-3.004 | 2.25E-03 | 5.70 | Up |
|  | Homoserine.18* | 3.859-3.855 | 1.82E-02 | 5.71 | Up |
|  | 2-Oxoglutarate.4*, 4-Aminobutyrate.8* | 3.004-2.992 | 9.42E-04 | 5.87 | Up |
|  | 4-Aminobutyrate.7* | 2.294-2.280 | 5.97E-04 | 6.04 | Up |
|  | Aspartate.8* | 2.697-2.687 | 5.06E-03 | 6.07 | Up |
|  | Isoleucine.12* | 3.670-3.662 | 4.72E-04 | 6.10 | Up |
|  | β-Alanine.5* | 2.561-2.556 | 8.94E-03 | 6.43 | Up |
|  | 2-Aminoadipate.10* | 2.270-2.265 | 6.14E-03 | 6.54 | Up |
|  | Homoserine.17*, Uridine.7* | 3.811-3.806 | 4.15E-03 | 6.91 | Up |
|  | Choline.10* | 4.044-4.039 | 4.72E-02 | 6.98 | Up |
|  | Agmatine.5* | 3.027-3.016 | 5.97E-04 | 6.99 | Up |
|  | 2-Aminoadipate.9* | 2.275-2.270 | 6.14E-03 | 7.04 | Up |
|  | Choline.9* | 3.195-3.186 | 3.40E-03 | 7.05 | Up |
|  | 2-Aminoadipate.8*, 4-Aminobutyrate.6*, Acetate* | 1.914-1.902 | 4.72E-04 | 7.15 | Up |
|  | 4-Aminobutyrate.5* | 2.280-2.275 | 8.94E-03 | 7.16 | Up |
|  | Ethanolamine.5*, Phenylalanine.16* | 3.134-3.060 | 1.28E-02 | 7.51 | Up |
| Left  Cerebrum | 2-Aminoadipate.7*, 4-Aminobutyrate.4* | 1.902-1.890 | 2.29E-04 | 7.57 | Up |
|  | Aspartate.7*, Homoserine.16* | 3.878-3.870 | 1.47E-03 | 7.96 | Up |
|  | Uridine.6* | 3.851-3.845 | 1.47E-03 | 8.01 | Up |
|  | Choline.8* | 3.524-3.511 | 1.28E-02 | 8.20 | Up |
|  | 2-Aminoadipate.6*, 4-Aminobutyrate.3* | 1.923-1.914 | 1.78E-04 | 8.37 | Up |
|  | Nicotinurate.3* | 3.971-3.963 | 4.15E-03 | 8.56 | Up |
|  | Isoleucine.11* | 1.432-1.423 | 9.42E-04 | 8.59 | Up |
|  | 2-Aminoadipate.5*, 4-Aminobutyrate.2* | 1.890-1.881 | 1.78E-04 | 8.64 | Up |
|  | Isoleucine.10* | 3.683-3.677 | 3.72E-04 | 9.11 | Up |
|  | Choline.7* | 3.538-3.532 | 5.06E-03 | 9.82 | Up |
|  | Uridine.5* | 3.919-3.912 | 5.97E-04 | 9.91 | Up |
|  | Alanine.6*, Homoserine.15* | 3.790-3.786 | 2.92E-04 | 9.92 | Up |
|  | β-Alanine.4* | 2.545-2.537 | 8.94E-03 | 9.94 | Up |
|  | Aspartate.6*, Uridine.4* | 3.905-3.900 | 2.29E-04 | 10.17 | Up |
|  | Choline.6* | 3.532-3.524 | 1.28E-02 | 10.22 | Up |
|  | Choline.5* | 3.186-3.178 | 3.72E-04 | 10.94 | Up |
|  | Alanine.5*, Homoserine.14* | 3.803-3.794 | 8.25E-05 | 11.11 | Up |
|  | 2-Aminoadipate.4*, 4-Aminobutyrate.1*, Glutaric Acid Monomethyl Ester.2 | 1.881-1.764 | 7.51E-04 | 11.28 | Up |
|  | Choline.4* | 3.511-3.492 | 2.98E-02 | 11.30 | Up |
|  | Histidine.13* | 3.217-3.195 | 8.94E-03 | 11.61 | Up |
|  | 2-Aminoadipate.3* | 3.757-3.750 | 1.38E-04 | 11.89 | Up |
|  | Isoleucine.9* | 1.423-1.414 | 3.72E-04 | 12.02 | Up |
| Left  Cerebrum | Aspartate.5* | 2.723-2.711 | 1.38E-04 | 12.21 | Up |
|  | Aspartate.4* | 2.733-2.723 | 1.07E-04 | 12.22 | Up |
|  | Homoserine.13* | 2.004-1.994 | 1.78E-04 | 12.25 | Up |
|  | β-Alanine.3* | 3.178-3.171 | 1.38E-04 | 12.42 | Up |
|  | Alanine.4* | 1.499-1.487 | 3.72E-04 | 12.62 | Up |
|  | Isoleucine.8* | 1.013-1.004 | 1.18E-03 | 12.93 | Up |
|  | Homoserine.12* | 1.938-1.923 | 1.18E-03 | 12.98 | Up |
|  | Alanine.3*, Homoserine.11* | 3.786-3.777 | 3.40E-03 | 13.01 | Up |
|  | Histidine.12* | 7.155-7.140 | 3.48E-02 | 13.04 | Up |
|  | β-Alanine.2* | 3.171-3.165 | 1.78E-04 | 13.42 | Up |
|  | Glutaric Acid Monomethyl Ester.1 | 3.712-3.702 | 8.25E-05 | 13.89 | Up |
|  | Alanine.2* | 1.487-1.474 | 1.38E-04 | 14.02 | Up |
|  | Uridine.3* | 3.845-3.839 | 7.42E-03 | 14.26 | Up |
|  | Isoleucine.7* | 3.687-3.683 | 8.25E-05 | 14.66 | Up |
|  | Histidine.11* | 3.338-3.315 | 1.18E-03 | 14.83 | Up |
|  | Histidine.10* | 4.012-4.007 | 1.07E-04 | 14.87 | Up |
|  | Homoserine.10* | 3.855-3.851 | 9.42E-04 | 15.28 | Up |
|  | 2-Aminobutyrate.5, Leucine.6* | 3.737-3.731 | 8.25E-05 | 15.65 | Up |
|  | 2-Aminobutyrate.4, Isoleucine.6*, Leucine.5* | 0.948-0.938 | 1.18E-03 | 15.83 | Up |
|  | 2-Aminobutyrate.3, Isoleucine.5*, Leucine.4* | 0.938-0.929 | 5.97E-04 | 15.86 | Up |
|  | Homoserine.9* | 1.975-1.938 | 1.38E-04 | 16.02 | Up |
|  | Histidine.9* | 4.007-4.003 | 8.25E-05 | 16.20 | Up |
| Left  Cerebrum | Taurine.3* | 3.397-3.391 | 2.92E-04 | 16.59 | Up |
|  | Homoserine.8* | 1.994-1.985 | 8.25E-05 | 16.78 | Up |
|  | Taurine.2* | 3.407-3.397 | 1.38E-04 | 17.40 | Up |
|  | Agmatine.4* | 2.831-2.823 | 8.25E-05 | 17.66 | Up |
|  | Histidine.8* | 4.025-4.021 | 8.25E-05 | 17.82 | Up |
|  | Choline.3* | 3.578-3.567 | 8.25E-05 | 17.96 | Up |
|  | Homoserine.7* | 1.985-1.975 | 1.07E-04 | 18.06 | Up |
|  | Isoleucine.4* | 1.004-0.995 | 1.07E-04 | 18.53 | Up |
|  | Aspartate.3* | 2.798-2.789 | 8.25E-05 | 18.83 | Up |
|  | 2-Aminoadipate.2* | 3.746-3.737 | 1.07E-04 | 18.97 | Up |
|  | Aspartate.2* | 2.811-2.798 | 8.25E-05 | 19.05 | Up |
|  | Niacinamide.2* | 8.238-8.224 | 8.25E-05 | 19.09 | Up |
|  | Hypoxanthine* | 8.224-8.211 | 8.25E-05 | 19.46 | Up |
|  | Histidine.7* | 4.021-4.012 | 8.25E-05 | 19.65 | Up |
|  | Ethanolamine.4* | 3.143-3.134 | 1.38E-04 | 19.93 | Up |
|  | Agmatine.3*, Leucine.3* | 1.764-1.640 | 1.38E-04 | 20.53 | Up |
|  | Ethanolamine.3*, Homoserine.6* | 3.824-3.818 | 8.25E-05 | 20.67 | Up |
|  | 3-Phenylpropionate.2* | 7.295-7.283 | 1.82E-03 | 21.03 | Up |
|  | Aspartate.1* | 2.823-2.811 | 8.25E-05 | 21.10 | Up |
|  | Histidine.6* | 3.353-3.344 | 8.25E-05 | 22.50 | Up |
|  | Homoserine.5* | 2.176-2.166 | 7.42E-03 | 23.11 | Up |
|  | Phenylalanine.15* | 7.377-7.368 | 2.29E-04 | 23.16 | Up |
|  | Phenylalanine.14* | 7.752-7.729 | 2.53E-02 | 23.89 | Up |
| Left  Cerebrum | Ethanolamine.2*, Homoserine.4* | 3.831-3.824 | 1.38E-04 | 24.52 | Up |
|  | 2-Aminobutyrate.2, Leucine.2* | 3.731-3.723 | 8.25E-05 | 24.65 | Up |
|  | 5,6-Dihydrouracil.3* | 2.660-2.650 | 8.25E-05 | 25.01 | Up |
|  | Uridine.2* | 3.839-3.831 | 1.78E-04 | 25.76 | Up |
|  | Histidine.5* | 3.360-3.353 | 8.25E-05 | 25.76 | Up |
|  | Alanine.1*, Homoserine.3* | 3.794-3.790 | 1.38E-04 | 26.43 | Up |
|  | Homoserine.2* | 2.166-2.129 | 2.77E-03 | 26.71 | Up |
|  | Phenylalanine.13* | 7.389-7.377 | 1.38E-04 | 26.94 | Up |
|  | Phenylalanine.12* | 7.352-7.337 | 1.07E-04 | 27.29 | Up |
|  | Histidine.4* | 3.344-3.338 | 8.25E-05 | 27.30 | Up |
|  | 2-Aminobutyrate.1, Leucine.1* | 0.980-0.948 | 8.25E-05 | 28.28 | Up |
|  | Phenylalanine.11* | 7.337-7.324 | 1.07E-04 | 28.33 | Up |
|  | Phenylalanine.10* | 7.554-7.549 | 1.07E-04 | 28.91 | Up |
|  | Histidine.3* | 7.199-7.185 | 8.25E-05 | 29.08 | Up |
|  | Phenylalanine.9* | 7.397-7.389 | 8.25E-05 | 29.70 | Up |
|  | Nicotinurate.2* | 8.699-8.683 | 2.53E-02 | 30.33 | Up |
|  | β-Alanine.1* | 3.159-3.152 | 8.25E-05 | 30.41 | Up |
|  | 3-Phenylpropionate.1* | 7.210-7.199 | 8.25E-05 | 30.45 | Up |
|  | Phenylalanine.8* | 6.916-6.903 | 8.25E-05 | 31.69 | Up |
|  | Phenylalanine.7* | 6.929-6.916 | 8.25E-05 | 31.75 | Up |
|  | Isoleucine.3* | 1.242-1.233 | 8.25E-05 | 32.23 | Up |
|  | Phenylalanine.6* | 3.152-3.143 | 1.07E-04 | 32.74 | Up |
|  | Phenylalanine.5* | 7.440-7.429 | 8.25E-05 | 33.28 | Up |
| Left  Cerebrum | 5,6-Dihydrouracil.2* | 2.639-2.633 | 8.25E-05 | 33.53 | Up |
|  | Histidine.2* | 8.085-8.069 | 8.25E-05 | 33.59 | Up |
|  | Niacinamide.1* | 8.712-8.699 | 9.42E-04 | 33.82 | Up |
|  | Phenylalanine.4* | 7.450-7.440 | 8.25E-05 | 34.10 | Up |
|  | Phenylalanine.3* | 7.429-7.418 | 8.25E-05 | 34.46 | Up |
|  | Valine.4* | 1.056-1.046 | 8.25E-05 | 35.81 | Up |
|  | Uracil.7* | 7.576-7.567 | 8.25E-05 | 35.85 | Up |
|  | 2-Oxoglutarate.3* | 2.439-2.429 | 1.47E-03 | 36.03 | Up |
|  | Valine.3* | 1.023-1.013 | 8.25E-05 | 36.31 | Up |
|  | Isoleucine.2*, Valine.2* | 0.995-0.985 | 8.25E-05 | 36.78 | Up |
|  | Phenylalanine.2* | 7.812-7.806 | 1.18E-03 | 38.09 | Up |
|  | 2-Oxoglutarate.2* | 2.452-2.439 | 9.42E-04 | 38.23 | Up |
|  | Uracil.6* | 7.567-7.558 | 8.25E-05 | 38.53 | Up |
|  | Valine.1* | 1.046-1.035 | 8.25E-05 | 38.58 | Up |
|  | Isoleucine.1* | 1.251-1.242 | 8.25E-05 | 38.70 | Up |
|  | Agmatine.2* | 3.249-3.241 | 8.25E-05 | 39.96 | Up |
|  | 5,6-Dihydrouracil.1* | 2.650-2.643 | 8.25E-05 | 40.54 | Up |
|  | Pyridoxine | 2.476-2.464 | 1.47E-03 | 41.27 | Up |
|  | 2-Oxoglutarate.1* | 2.464-2.452 | 1.47E-03 | 41.41 | Up |
|  | Choline.2* | 3.480-3.468 | 1.38E-04 | 41.70 | Up |
|  | Ethanolamine.1*, Homoserine.1*, Uridine.1* | 3.818-3.811 | 8.25E-05 | 41.81 | Up |
|  | Choline.1* | 3.492-3.480 | 1.38E-04 | 42.05 | Up |
| Left  Cerebrum | Histidine.1* | 7.166-7.155 | 8.25E-05 | 44.49 | Up |
|  | Nicotinurate.1* | 8.561-8.547 | 8.25E-05 | 44.55 | Up |
|  | Uracil.5* | 5.809-5.803 | 8.25E-05 | 51.82 | Up |
|  | Uracil.4* | 5.820-5.814 | 8.25E-05 | 54.35 | Up |
|  | Agmatine.1*, Phenylalanine.1*, Taurine.1* | 3.268-3.249 | 8.25E-05 | 62.38 | Up |
|  | 2-Aminoadipate.1* | 2.243-2.234 | 8.25E-05 | 68.96 | Up |
|  | Uracil.3* | 5.414-5.406 | 1.82E-02 | 200.28 | Up |
|  | Uracil.2* | 5.258-5.253 | 1.53E-02 | 420.13 | Up |
|  | Uracil.1* | 5.253-5.248 | 1.53E-02 | 503.57 | Up |
| Right Cerebrum | Inosine.1 | 6.119-6.108 | 8.25E-05 | -108.20 | Down |
|  | Inosine.2 | 6.108-6.097 | 8.25E-05 | -106.69 | Down |
|  | Adenosine.1*, Inosine.3 | 8.378-8.354 | 8.25E-05 | -102.02 | Down |
|  | Nicotinate.1 | 8.268-8.259 | 8.25E-05 | -98.94 | Down |
|  | Methylmalonate.1 | 1.194-1.185 | 8.25E-05 | -98.38 | Down |
|  | Inosine.4 | 6.097-6.088 | 8.25E-05 | -87.98 | Down |
|  | N-Acetylaspartate.1* | 7.992-7.946 | 8.25E-05 | -79.16 | Down |
|  | Guanosine.1 | 4.457-4.449 | 8.25E-05 | -78.45 | Down |
|  | Adenosine.2* | 6.088-6.074 | 1.07E-04 | -76.58 | Down |
|  | N-Acetylaspartate.2* | 2.681-2.677 | 8.25E-05 | -76.09 | Down |
|  | N-Acetylaspartate.3* | 4.398-4.389 | 8.25E-05 | -72.80 | Down |
|  | Glutamate.1* | 2.358-2.348 | 8.25E-05 | -72.21 | Down |
|  | Glutamate.2* | 2.348-2.336 | 8.25E-05 | -71.45 | Down |
| Right  Cerebrum | N-Acetylaspartate.4* | 2.687-2.681 | 8.25E-05 | -70.93 | Down |
|  | N-Acetylaspartate.5* | 4.389-4.384 | 8.25E-05 | -70.20 | Down |
|  | N-Acetylaspartate.6* | 4.384-4.379 | 8.25E-05 | -68.17 | Down |
|  | Adenosine.3* | 4.290-4.284 | 8.25E-05 | -66.48 | Down |
|  | Glutamate.3* | 2.336-2.328 | 8.25E-05 | -64.66 | Down |
|  | Glutamate.4*, N-Acetylaspartate.7*, Pyroglutamate.1* | 2.035-2.013 | 8.25E-05 | -62.80 | Down |
|  | Singlet 1.178 ppm | 1.185-1.171 | 8.25E-05 | -61.98 | Down |
|  | Inosine.5, N-Acetylaspartate.8* | 4.379-4.373 | 8.25E-05 | -61.45 | Down |
|  | Adenosine.4* | 4.405-4.398 | 8.25E-05 | -61.37 | Down |
|  | Carnitine.1* | 4.463-4.457 | 8.25E-05 | -61.15 | Down |
|  | π-Methylhistidine | 8.278-8.268 | 8.25E-05 | -60.98 | Down |
|  | Glutamate.5*, Glycerol.1* | 3.772-3.766 | 8.25E-05 | -55.32 | Down |
|  | Glutamate.6* | 2.129-2.119 | 8.25E-05 | -55.27 | Down |
|  | Guanosine.2 | 4.449-4.439 | 8.25E-05 | -55.16 | Down |
|  | Glutamate.7*, Glycerol.2* | 3.766-3.757 | 8.25E-05 | -54.81 | Down |
|  | Glutamate.8* | 2.372-2.362 | 8.25E-05 | -54.43 | Down |
|  | Adenosine.5* | 4.410-4.405 | 8.25E-05 | -53.71 | Down |
|  | Glutamate.9* | 2.119-2.107 | 8.25E-05 | -52.82 | Down |
|  | Adenosine.6* | 4.284-4.274 | 8.25E-05 | -52.46 | Down |
|  | N-Acetylaspartate.9* | 7.946-7.932 | 8.25E-05 | -52.18 | Down |
|  | N-Acetylaspartate.10*, Pyroglutamate.2* | 2.514-2.507 | 8.25E-05 | -49.68 | Down |
|  | Guanosine.3, Histamine.1 | 8.020-8.014 | 8.25E-05 | -49.34 | Down |
| Right  Cerebrum | Fumarate* | 6.163-6.119 | 2.29E-04 | -49.10 | Down |
|  | Methylmalonate.2 | 1.205-1.194 | 8.25E-05 | -49.06 | Down |
|  | Nicotinate.2 | 8.288-8.278 | 8.25E-05 | -48.44 | Down |
|  | 1,3-Dimethylurate.1* | 3.241-3.232 | 8.25E-05 | -46.63 | Down |
|  | Glutamate.10* | 2.085-2.075 | 8.25E-05 | -46.57 | Down |
|  | Inosine.6 | 4.295-4.290 | 8.25E-05 | -44.44 | Down |
|  | Pyroglutamate.3* | 2.533-2.521 | 8.25E-05 | -42.82 | Down |
|  | Glutamate.11*, Glycerol.3* | 3.777-3.772 | 8.25E-05 | -41.23 | Down |
|  | Levulinate.1*, Pyroglutamate.4* | 2.379-2.372 | 8.25E-05 | -40.10 | Down |
|  | Adenosine.7* | 6.009-5.938 | 9.42E-04 | -38.78 | Down |
|  | N-Acetylaspartate.11*, Pyroglutamate.5* | 2.507-2.498 | 2.25E-03 | -37.83 | Down |
|  | N-Acetylaspartate.12*, Pyroglutamate.6* | 2.498-2.485 | 5.97E-04 | -37.61 | Down |
|  | D-Threitol.1* | 3.695-3.691 | 8.25E-05 | -37.12 | Down |
|  | Glutamate.12* | 2.094-2.085 | 8.25E-05 | -35.97 | Down |
|  | Adenosine.8*, Creatine*, Tyrosine.1* | 3.931-3.919 | 8.25E-05 | -35.42 | Down |
|  | D-Threitol.2* | 3.691-3.687 | 8.25E-05 | -34.98 | Down |
|  | Nicotinate.3 | 8.608-8.593 | 1.82E-03 | -34.41 | Down |
|  | Glutamate.13* | 2.107-2.100 | 8.25E-05 | -31.62 | Down |
|  | Glutamate.14* | 2.321-2.317 | 8.25E-05 | -31.16 | Down |
|  | N-Acetylaspartate.13* | 2.711-2.697 | 8.25E-05 | -30.29 | Down |
|  | D-Threitol.3* | 3.699-3.695 | 8.25E-05 | -30.17 | Down |
|  | Adenosine.9* | 8.259-8.249 | 8.25E-05 | -30.17 | Down |
|  | Glutamate.15* | 2.075-2.064 | 8.25E-05 | -29.30 | Down |
| Right  Cerebrum | Formate* | 8.475-8.453 | 8.25E-05 | -28.94 | Down |
|  | Glutamate.16* | 2.064-2.056 | 8.25E-05 | -27.23 | Down |
|  | D-Threitol.4* | 3.702-3.699 | 8.25E-05 | -25.88 | Down |
|  | Adenine.1 | 8.148-8.136 | 8.25E-05 | -25.70 | Down |
|  | Glutamate.17* | 2.325-2.321 | 8.25E-05 | -25.15 | Down |
|  | Carnitine.2*, Levulinate.2*, Pyroglutamate.7* | 2.391-2.385 | 8.25E-05 | -24.72 | Down |
|  | Guanosine.4 | 3.996-3.989 | 8.25E-05 | -24.40 | Down |
|  | Adenosine.10*, Inosine.7 | 4.300-4.295 | 8.25E-05 | -23.14 | Down |
|  | Myo-Inositol.1* | 3.278-3.268 | 1.78E-04 | -22.91 | Down |
|  | Adenosine.11*, Inosine.8 | 4.304-4.300 | 8.25E-05 | -22.81 | Down |
|  | Glutamate.18* | 2.362-2.358 | 8.25E-05 | -22.40 | Down |
|  | Creatine Phosphate | 3.060-3.049 | 7.51E-04 | -22.38 | Down |
|  | Lactate.1* | 4.114-4.106 | 8.25E-05 | -22.17 | Down |
|  | Pyroglutamate.8* | 4.125-4.114 | 8.25E-05 | -21.40 | Down |
|  | Glutamate.19*, Glycerol.4* | 3.786-3.777 | 2.25E-03 | -21.26 | Down |
|  | Myo-Inositol.2* | 3.289-3.278 | 5.97E-04 | -20.70 | Down |
|  | Lactate.2* | 4.106-4.101 | 8.25E-05 | -20.69 | Down |
|  | Carnitine.3* | 3.428-3.417 | 3.72E-04 | -20.29 | Down |
|  | 1,3-Dimethylurate.2* | 3.441-3.428 | 2.29E-04 | -19.64 | Down |
|  | Adenosine.12*, Tyrosine.2* | 3.947-3.931 | 8.25E-05 | -19.47 | Down |
|  | Histamine.2 | 8.014-7.992 | 1.38E-04 | -19.27 | Down |
|  | 1,3-Dimethylurate.3*, Myo-Inositol.3* | 3.304-3.289 | 1.47E-03 | -18.98 | Down |
| Right  Cerebrum | N-Acetylaspartate.14* | 2.669-2.664 | 8.25E-05 | -18.95 | Down |
|  | Creatinine.1* | 3.049-3.033 | 8.25E-05 | -18.55 | Down |
|  | Inosine.9, N-Acetylaspartate.15* | 4.373-4.368 | 4.72E-04 | -17.67 | Down |
|  | Guanosine.5 | 3.989-3.980 | 3.72E-04 | -17.26 | Down |
|  | Carnitine.4* | 3.417-3.407 | 1.18E-03 | -16.83 | Down |
|  | Caprate.1 | 0.804-0.793 | 7.42E-03 | -16.32 | Down |
|  | Glutamate.20* | 2.166-2.129 | 3.40E-03 | -16.14 | Down |
|  | Nicotinate.4 | 8.963-8.940 | 1.38E-04 | -15.97 | Down |
|  | 3-Hydroxybutyrate.1 | 1.113-1.106 | 3.72E-04 | -15.84 | Down |
|  | D-Threitol.5* | 3.683-3.677 | 8.25E-05 | -15.64 | Down |
|  | Lactate.3*, Threonine.1* | 1.351-1.311 | 1.78E-04 | -15.37 | Down |
|  | Pyroglutamate.9* | 4.136-4.125 | 8.25E-05 | -15.00 | Down |
|  | Guanosine.6 | 5.924-5.917 | 3.40E-03 | -14.89 | Down |
|  | Lactate.4* | 4.098-4.093 | 1.38E-04 | -14.81 | Down |
|  | N-Acetylaspartate.16* | 2.664-2.660 | 2.29E-04 | -14.79 | Down |
|  | Serine.1* | 4.031-4.025 | 8.25E-05 | -13.90 | Down |
|  | Threonine.2* | 4.230-4.218 | 4.72E-02 | -13.63 | Down |
|  | Caprate.2 | 0.793-0.780 | 4.15E-03 | -13.62 | Down |
|  | Glycerol.5*, Myo-Inositol.4* | 3.550-3.538 | 1.47E-03 | -13.60 | Down |
|  | Glycerol.6* | 3.647-3.638 | 1.47E-03 | -12.92 | Down |
|  | D-Threitol.6*, Glycerol.7*, Myo-Inositol.5* | 3.638-3.622 | 1.47E-03 | -12.90 | Down |
|  | 3-Hydroxybutyrate.2 | 1.080-1.061 | 2.92E-04 | -12.86 | Down |
| Right  Cerebrum | Threonine.3* | 4.240-4.230 | 5.06E-03 | -12.64 | Down |
|  | Pyroglutamate.10* | 2.556-2.551 | 3.72E-04 | -12.63 | Down |
|  | Indole-3-Acetate.1 | 7.611-7.605 | 9.42E-04 | -12.52 | Down |
|  | Xanthurenate.1 | 7.616-7.611 | 9.42E-04 | -11.65 | Down |
|  | Nicotinate.5 | 8.735-8.723 | 2.92E-04 | -11.44 | Down |
|  | Carnitine.5* | 3.315-3.304 | 1.47E-03 | -10.76 | Down |
|  | D-Threitol.7* | 3.719-3.712 | 1.38E-04 | -10.64 | Down |
|  | Serine.2* | 4.039-4.031 | 1.78E-04 | -10.48 | Down |
|  | Guanosine.7 | 5.913-5.906 | 2.15E-02 | -10.16 | Down |
|  | Nicotinate.6 | 8.723-8.712 | 3.72E-04 | -9.93 | Down |
|  | Inosine.10, N-Acetylaspartate.17* | 4.368-4.359 | 1.82E-02 | -9.92 | Down |
|  | Adenosine.13*, Serine.3* | 3.845-3.839 | 9.42E-04 | -9.83 | Down |
|  | Doublet 3.881 ppm | 3.883-3.878 | 1.38E-04 | -9.55 | Down |
|  | D-Threitol.8*, Myo-Inositol.6* | 3.622-3.611 | 1.47E-03 | -9.53 | Down |
|  | Indole-3-Acetate.2 | 7.623-7.616 | 1.47E-03 | -9.25 | Down |
|  | Xanthurenate.2 | 7.605-7.598 | 5.97E-04 | -9.12 | Down |
|  | Carnitine.6* | 3.391-3.370 | 9.42E-04 | -9.07 | Down |
|  | D-Threitol.9* | 3.687-3.683 | 2.25E-03 | -8.98 | Down |
|  | Caprate.3 | 2.187-2.176 | 3.40E-03 | -8.63 | Down |
|  | 4-Pyridoxate* | 7.898-7.890 | 1.07E-02 | -8.56 | Down |
|  | Glycerol.8* | 3.563-3.550 | 1.47E-03 | -8.21 | Down |
|  | Caprate.4 | 0.831-0.804 | 3.40E-03 | -8.09 | Down |
|  | Levulinate.3* | 2.868-2.862 | 8.94E-03 | -8.01 | Down |
| Right  Cerebrum | Adenine.2, Inosine.11 | 8.211-8.206 | 2.98E-02 | -6.96 | Down |
|  | Levulinate.4* | 2.943-2.931 | 1.82E-03 | -6.11 | Down |
|  | Carnitine.7*, Levulinate.5*, Pyroglutamate.11* | 2.385-2.379 | 1.28E-02 | -5.98 | Down |
|  | Methylamine | 2.607-2.603 | 1.82E-02 | -5.68 | Down |
|  | Creatinine.2* | 4.051-4.044 | 4.72E-02 | -5.61 | Down |
|  | Levulinate.6* | 2.243-2.234 | 2.25E-03 | -5.17 | Down |
|  | Lactate.5* | 4.101-4.098 | 4.06E-02 | -5.12 | Down |
|  | Myo-Inositol.7* | 4.093-4.055 | 2.98E-02 | -4.45 | Down |
|  | Glycerol.9* | 3.670-3.662 | 1.82E-02 | -4.43 | Down |
|  | Doublet 3.865 ppm | 3.870-3.859 | 7.42E-03 | -4.37 | Down |
|  | Levulinate.7* | 2.918-2.868 | 2.15E-02 | -3.63 | Down |
|  | Uridine.8* | 3.912-3.905 | 3.48E-02 | 2.02 | Up |
|  | N-Acetylornithine.6 | 4.148-4.136 | 2.98E-02 | 2.62 | Up |
|  | Aspartate.11* | 2.755-2.733 | 4.06E-02 | 2.98 | Up |
|  | Homocysteine.14 | 2.223-2.216 | 2.25E-03 | 4.56 | Up |
|  | β-Alanine.6* | 2.561-2.556 | 2.25E-03 | 5.19 | Up |
|  | Homocysteine.13, Valine.5* | 2.255-2.243 | 2.77E-03 | 5.59 | Up |
|  | Phenylalanine.11* | 3.134-3.060 | 7.42E-03 | 6.14 | Up |
|  | Homocysteine.12, Homoserine.16* | 3.878-3.870 | 1.07E-02 | 7.15 | Up |
|  | Histidine.10* | 3.338-3.315 | 1.28E-02 | 7.28 | Up |
|  | Aspartate.10*, Homocysteine.11 | 2.677-2.672 | 1.18E-03 | 7.35 | Up |
|  | Isoleucine.17* | 1.251-1.242 | 1.28E-02 | 8.00 | Up |
| Right  Cerebrum | 5,6-Dihydrouracil.6* | 3.468-3.464 | 7.51E-04 | 8.79 | Up |
|  | Isoleucine.16* | 3.653-3.647 | 5.97E-04 | 8.88 | Up |
|  | Homocysteine.10, Isoleucine.15* | 1.242-1.233 | 2.92E-04 | 9.33 | Up |
|  | β-Alanine.5* | 2.571-2.565 | 8.25E-05 | 9.53 | Up |
|  | Homoserine.15* | 3.839-3.831 | 8.94E-03 | 9.73 | Up |
|  | Saccharopine.3 | 3.591-3.585 | 3.72E-04 | 9.94 | Up |
|  | N-Acetylornithine.5 | 4.159-4.155 | 4.06E-02 | 10.63 | Up |
|  | 2-Oxoglutarate.2* | 2.429-2.415 | 4.72E-02 | 11.24 | Up |
|  | Alanine.6*, Homoserine.14* | 3.794-3.790 | 1.53E-02 | 11.57 | Up |
|  | Homoserine.13* | 3.960-3.953 | 1.07E-04 | 11.76 | Up |
|  | Homoserine.12* | 2.045-2.035 | 2.25E-03 | 12.12 | Up |
|  | N-Acetylornithine.4 | 4.162-4.159 | 8.94E-03 | 12.26 | Up |
|  | Homoserine.11* | 3.963-3.960 | 1.38E-04 | 12.37 | Up |
|  | Choline.8* | 4.044-4.039 | 7.51E-04 | 12.66 | Up |
|  | Agmatine.7*, Phenylalanine.10* | 3.268-3.249 | 8.25E-05 | 13.59 | Up |
|  | Isoleucine.14* | 1.214-1.205 | 2.29E-04 | 13.64 | Up |
|  | Ethanolamine.6*, Homoserine.10*, Uridine.7* | 3.824-3.818 | 8.25E-05 | 13.96 | Up |
|  | Choline.7* | 3.195-3.186 | 8.25E-05 | 14.02 | Up |
|  | Isoleucine.13* | 3.677-3.670 | 1.07E-04 | 14.86 | Up |
|  | Agmatine.6* | 3.232-3.217 | 1.07E-02 | 14.88 | Up |
|  | Ethanolamine.5* | 3.165-3.159 | 2.29E-04 | 15.06 | Up |
|  | Alanine.5* | 3.790-3.786 | 8.25E-05 | 15.74 | Up |
| Right  Cerebrum | N-Acetylornithine.3 | 4.166-4.162 | 2.25E-03 | 16.02 | Up |
|  | Homoserine.9* | 3.831-3.824 | 1.78E-04 | 16.09 | Up |
|  | Uridine.6* | 3.851-3.845 | 8.25E-05 | 16.87 | Up |
|  | Taurine.5* | 3.397-3.391 | 8.25E-05 | 16.88 | Up |
|  | N-Acetylornithine.2 | 4.181-4.166 | 5.06E-03 | 17.26 | Up |
|  | Choline.6 * | 3.524-3.511 | 1.07E-04 | 18.23 | Up |
|  | S-Adenosylhomocysteine.4 | 4.274-4.264 | 3.72E-04 | 19.31 | Up |
|  | 4-Aminobutyrate.10* | 1.881-1.764 | 1.07E-04 | 19.50 | Up |
|  | Choline.5* | 3.538-3.532 | 2.29E-04 | 20.08 | Up |
|  | 3-Phenylpropionate.6* | 7.283-7.273 | 1.07E-04 | 20.38 | Up |
|  | Taurine.4* | 3.370-3.360 | 1.53E-02 | 20.64 | Up |
|  | 2-Aminoadipate.6* | 3.757-3.750 | 8.25E-05 | 20.89 | Up |
|  | Alanine.4* | 3.806-3.803 | 8.25E-05 | 21.12 | Up |
|  | Nicotinurate.2* | 8.699-8.683 | 7.51E-04 | 21.50 | Up |
|  | Adenosine Monophosphate | 8.561-8.547 | 2.25E-03 | 21.66 | Up |
|  | Homoserine.8* | 3.980-3.971 | 8.25E-05 | 21.81 | Up |
|  | Saccharopine.2 | 3.596-3.591 | 8.25E-05 | 21.85 | Up |
|  | Tryptophan.4 | 7.752-7.729 | 6.14E-03 | 21.85 | Up |
|  | Aspartate.9* | 2.697-2.687 | 8.25E-05 | 21.93 | Up |
|  | Aspartate.8*, Uridine.5* | 3.900-3.883 | 8.25E-05 | 22.16 | Up |
|  | 2-Aminoadipate.5* | 2.265-2.255 | 8.25E-05 | 22.29 | Up |
|  | Histidine.9* | 4.003-3.996 | 8.25E-05 | 22.56 | Up |
| Right  Cerebrum | Choline.4* | 3.567-3.563 | 8.25E-05 | 22.76 | Up |
|  | β-Alanine.3* | 3.186-3.178 | 8.25E-05 | 22.99 | Up |
|  | β-Alanine.4* | 3.178-3.171 | 8.25E-05 | 22.99 | Up |
|  | Homocysteine.9, Isoleucine.12* | 1.432-1.423 | 8.25E-05 | 23.02 | Up |
|  | S-Adenosylhomocysteine.3 | 4.439-4.430 | 7.51E-04 | 23.07 | Up |
|  | Leucine.4* | 3.731-3.723 | 8.25E-05 | 23.22 | Up |
|  | S-Adenosylhomocysteine.2 | 4.255-4.247 | 1.38E-04 | 23.67 | Up |
|  | 3-Phenylpropionate.5* | 7.295-7.283 | 8.25E-05 | 23.71 | Up |
|  | Ethanolamine.4*, Homoserine.7*, Uridine.4* | 3.818-3.811 | 8.25E-05 | 23.78 | Up |
|  | Saccharopine.1 | 3.585-3.578 | 8.25E-05 | 24.15 | Up |
|  | Histidine.8* | 7.932-7.921 | 8.25E-05 | 24.56 | Up |
|  | Nicotinurate.1* | 3.953-3.947 | 8.25E-05 | 24.84 | Up |
|  | Ethanolamine.3* | 3.143-3.134 | 8.25E-05 | 24.90 | Up |
|  | Isoleucine.11* | 0.895-0.890 | 8.25E-05 | 25.51 | Up |
|  | Choline.3* | 3.532-3.524 | 1.38E-04 | 25.70 | Up |
|  | 2-Aminoadipate.4*, Leucine.3* | 3.737-3.731 | 8.25E-05 | 26.07 | Up |
|  | Taurine.3* | 3.407-3.397 | 8.25E-05 | 26.17 | Up |
|  | Aspartate.7 * | 2.768-2.755 | 8.25E-05 | 26.30 | Up |
|  | Homoserine.6* | 2.013-2.004 | 8.25E-05 | 26.84 | Up |
|  | Homocysteine.8 | 2.317-2.306 | 8.25E-05 | 27.09 | Up |
|  | 2-Aminoadipate.3* | 3.746-3.737 | 8.25E-05 | 27.46 | Up |
| Right  Cerebrum | Alanine.3*, Homoserine.5* | 3.803-3.794 | 8.25E-05 | 27.49 | Up |
|  | 3-Phenylpropionate.4*, Phenylalanine.9* | 7.304-7.295 | 2.29E-04 | 27.97 | Up |
|  | β-Alanine.2* | 3.171-3.165 | 8.25E-05 | 28.26 | Up |
|  | Uridine.3* | 3.919-3.912 | 8.25E-05 | 28.48 | Up |
|  | Uridine.2* | 3.855-3.851 | 8.25E-05 | 28.91 | Up |
|  | Valine.4* | 3.611-3.603 | 8.25E-05 | 29.11 | Up |
|  | Homocysteine.7 | 1.423-1.414 | 8.25E-05 | 29.59 | Up |
|  | Ethanolamine.2*, Homoserine.4* | 3.811-3.806 | 8.25E-05 | 29.91 | Up |
|  | β-Alanine.1* | 2.545-2.537 | 1.38E-04 | 30.25 | Up |
|  | Niacinamide.3* | 8.712-8.699 | 2.29E-04 | 30.72 | Up |
|  | 5,6-Dihydrouracil.5* | 3.480-3.468 | 1.78E-04 | 32.46 | Up |
|  | 5,6-Dihydrouracil.4* | 3.492-3.480 | 1.38E-04 | 33.41 | Up |
|  | 2'-Deoxyadenosine | 8.403-8.387 | 8.25E-05 | 33.53 | Up |
|  | 2-Oxoglutarate.1*, 4-Aminobutyrate.9* | 3.004-2.977 | 8.25E-05 | 33.69 | Up |
|  | Niacinamide.2* | 7.556-7.549 | 8.25E-05 | 33.81 | Up |
|  | 2-Aminoadipate.2* | 2.275-2.270 | 8.25E-05 | 34.36 | Up |
|  | Homoserine.3*, Isoleucine.10* | 2.004-1.994 | 8.25E-05 | 34.54 | Up |
|  | 2-Aminoadipate.1* | 2.270-2.265 | 8.25E-05 | 34.78 | Up |
|  | Aspartate.6*, Uridine.1* | 3.905-3.900 | 8.25E-05 | 34.79 | Up |
|  | Agmatine.5* | 3.027-3.016 | 8.25E-05 | 35.05 | Up |
|  | 4-Aminobutyrate.8* | 1.890-1.881 | 8.25E-05 | 35.25 | Up |
|  | Homocysteine.6 | 1.414-1.351 | 8.25E-05 | 35.30 | Up |
| Right  Cerebrum | Isoleucine.9* | 1.474-1.432 | 8.25E-05 | 35.45 | Up |
|  | Tryptophan.3 | 7.812-7.806 | 1.38E-04 | 35.62 | Up |
|  | Histidine.7* | 3.217-3.195 | 8.25E-05 | 35.64 | Up |
|  | N-Acetylornithine.1 | 4.155-4.148 | 8.25E-05 | 35.64 | Up |
|  | 4-Aminobutyrate.7*, Agmatine.4* | 3.016-3.004 | 8.25E-05 | 35.67 | Up |
|  | Homoserine.2* | 3.971-3.963 | 8.25E-05 | 35.82 | Up |
|  | Agmatine.3* | 3.033-3.027 | 8.25E-05 | 36.06 | Up |
|  | 4-Aminobutyrate.6* | 2.306-2.294 | 8.25E-05 | 36.36 | Up |
|  | 4-Aminobutyrate.5* | 2.294-2.280 | 8.25E-05 | 37.05 | Up |
|  | Choline.2* | 4.025-4.021 | 8.25E-05 | 37.05 | Up |
|  | S-Adenosylhomocysteine.1 | 4.264-4.255 | 8.25E-05 | 37.18 | Up |
|  | 4-Aminobutyrate.4* | 2.280-2.275 | 8.25E-05 | 37.49 | Up |
|  | 4-Aminobutyrate.3* | 1.902-1.890 | 8.25E-05 | 37.80 | Up |
|  | Tyramine.6 | 2.836-2.823 | 8.25E-05 | 38.02 | Up |
|  | Valine.3* | 3.603-3.596 | 8.25E-05 | 38.14 | Up |
|  | Aspartate.5*, Tyramine.5 | 2.823-2.811 | 8.25E-05 | 38.18 | Up |
|  | 4-Aminobutyrate.2* | 1.914-1.902 | 8.25E-05 | 39.09 | Up |
|  | 5,6-Dihydrouracil.3* | 2.639-2.633 | 8.25E-05 | 39.39 | Up |
|  | Aspartate.4* | 2.723-2.711 | 8.25E-05 | 39.47 | Up |
|  | Taurine.2* | 3.353-3.344 | 8.25E-05 | 39.53 | Up |
|  | Agmatine.2* | 3.249-3.241 | 8.25E-05 | 39.53 | Up |
|  | Tyramine.4 | 3.152-3.143 | 8.25E-05 | 39.93 | Up |
| Right  Cerebrum | Ethanolamine.1* | 3.159-3.152 | 8.25E-05 | 40.24 | Up |
|  | Isoleucine.8* | 1.013-1.004 | 8.25E-05 | 40.31 | Up |
|  | Histidine.6* | 4.012-4.007 | 8.25E-05 | 40.65 | Up |
|  | Aspartate.3* | 2.733-2.723 | 8.25E-05 | 41.31 | Up |
|  | 4-Aminobutyrate.1*, Acetate* | 1.923-1.914 | 8.25E-05 | 42.04 | Up |
|  | Aspartate.2*, Tyramine.3 | 2.811-2.798 | 8.25E-05 | 42.15 | Up |
|  | Histidine.5* | 3.344-3.338 | 8.25E-05 | 42.53 | Up |
|  | Histidine.4* | 4.021-4.012 | 8.25E-05 | 42.94 | Up |
|  | Aspartate.1* | 2.798-2.789 | 8.25E-05 | 43.36 | Up |
|  | 5,6-Dihydrouracil.2* | 2.660-2.650 | 8.25E-05 | 45.37 | Up |
|  | Homoserine.1*, Isoleucine.7* | 1.994-1.985 | 8.25E-05 | 45.42 | Up |
|  | Histidine.3* | 4.007-4.003 | 8.25E-05 | 46.02 | Up |
|  | Homocysteine.5, Isoleucine.6* | 1.975-1.938 | 8.25E-05 | 46.04 | Up |
|  | Homocysteine.4, Isoleucine.5* | 1.985-1.975 | 8.25E-05 | 46.37 | Up |
|  | Choline.1* | 3.578-3.567 | 8.25E-05 | 49.32 | Up |
|  | Alanine.2* | 1.499-1.487 | 8.25E-05 | 50.54 | Up |
|  | Alanine.1*, Isoleucine.4* | 1.487-1.474 | 8.25E-05 | 51.39 | Up |
|  | Taurine.1* | 3.360-3.353 | 8.25E-05 | 52.22 | Up |
|  | 5,6-Dihydrouracil.1* | 2.650-2.643 | 8.25E-05 | 53.50 | Up |
|  | Isoleucine.3* | 1.004-0.995 | 8.25E-05 | 54.99 | Up |
|  | Agmatine.1*, Leucine.2* | 1.764-1.621 | 8.25E-05 | 57.60 | Up |
|  | Homocysteine.3 | 1.023-1.013 | 8.25E-05 | 58.58 | Up |
| Right  Cerebrum | Isoleucine.2* | 0.938-0.929 | 8.25E-05 | 61.25 | Up |
|  | 3-Phenylpropionate.3*, Phenylalanine.8* | 7.377-7.368 | 8.25E-05 | 61.71 | Up |
|  | Tyramine.2 | 6.929-6.916 | 8.25E-05 | 62.80 | Up |
|  | Histidine.2* | 8.085-8.069 | 8.25E-05 | 62.90 | Up |
|  | Uracil.4* | 7.567-7.556 | 8.25E-05 | 63.38 | Up |
|  | Uracil.3* | 7.576-7.567 | 8.25E-05 | 64.74 | Up |
|  | Tyramine.1 | 6.916-6.903 | 8.25E-05 | 65.19 | Up |
|  | Niacinamide.1* | 8.238-8.224 | 8.25E-05 | 69.94 | Up |
|  | 3-Phenylpropionate.2*, Phenylalanine.7* | 7.389-7.377 | 8.25E-05 | 70.96 | Up |
|  | Phenylalanine.6* | 7.450-7.440 | 8.25E-05 | 71.33 | Up |
|  | Isoleucine.1* | 0.948-0.938 | 8.25E-05 | 71.40 | Up |
|  | Valine.2* | 0.995-0.985 | 8.25E-05 | 71.75 | Up |
|  | Hypoxanthine* | 8.224-8.211 | 8.25E-05 | 71.80 | Up |
|  | Homocysteine.2 | 1.938-1.923 | 8.25E-05 | 71.96 | Up |
|  | Histidine.1* | 7.199-7.185 | 8.25E-05 | 73.98 | Up |
|  | Phenylalanine.5* | 7.397-7.389 | 8.25E-05 | 74.69 | Up |
|  | Valine.1* | 1.046-1.035 | 8.25E-05 | 76.26 | Up |
|  | Homocysteine.1 | 1.056-1.046 | 8.25E-05 | 76.53 | Up |
|  | Tryptophan.2 | 7.210-7.199 | 8.25E-05 | 78.38 | Up |
|  | Tryptophan.1 | 7.166-7.155 | 8.25E-05 | 80.51 | Up |
|  | 3-Phenylpropionate.1*, Phenylalanine.4* | 7.337-7.324 | 8.25E-05 | 82.12 | Up |
|  | Phenylalanine.3* | 7.429-7.418 | 8.25E-05 | 82.90 | Up |
|  | Phenylalanine.2* | 7.352-7.337 | 8.25E-05 | 85.08 | Up |
| Right  Cerebrum | Leucine.1* | 0.980-0.948 | 8.25E-05 | 86.52 | Up |
|  | Phenylalanine.1* | 7.440-7.429 | 8.25E-05 | 86.76 | Up |
|  | Uracil.2* | 5.809-5.803 | 8.25E-05 | 90.99 | Up |
|  | Uracil.1* | 5.820-5.814 | 8.25E-05 | 91.81 | Up |
