# Supplementary material for: Early life stress shifts critical periods and causes precocious visual cortex development
Source: PLoS One. 2024 Dec 31;19(12):e0316384. doi: 10.1371/journal.pone.0316384 (PMC11687811; doi:10.1371/journal.pone.0316384)
Supplement: S3 Table — Metabolites found to be uniquely altered in either the left or right cerebral hemisphere using a Mann-Whitney U test. Metabolite regulation is shown as a function of relative concentration in high-EPS animals. Where more than one NMR resonance peak was identified as significant, the magnitude of change reported is the average percent difference of all relevant NMR resonance peaks. (DOCX) [file pone.0316384.s003.docx]

**Supplemental Table 3.** Cerebrum metabolites found to be significantly altered by stress in a lateralized manner. Metabolites found to be uniquely altered in either the left or right cerebral hemisphere using a Mann-Whitney U test. Metabolite regulation is shown as a function of relative concentration in high-EPS individuals. Where more than one NMR resonance peak was identified as significant, the magnitude of change reported is the average percent difference of all relevant NMR resonance peaks.

| **Region** | **Metabolite** | **Percent Difference** | **Regulation by Stress** |
| --- | --- | --- | --- |
| Left Cerebrum | 2-Aminobutyrate | 20.05 | Up |
|  | Glutaric Acid Monomethyl Ester | 12.59 | Up |
|  | Glycine | -4.89 | Down |
|  | O-Phosphocholine | -14.39 | Down |
|  | Pyridoxine | 41.27 | Up |
| Right Cerebrum | 2'-Deoxyadenosine | 33.53 | Up |
|  | 3-Hydroxybutyric acid | -14.35 | Down |
|  | Adenine | -16.33 | Down |
|  | Adenosine monophosphate | 21.66 | Up |
|  | Capric acid | -11.67 | Down |
|  | Phosphocreatine | -22.38 | Down |
|  | Guanosine | -35.67 | Down |
|  | Histamine | -34.31 | Down |
|  | Homocysteine | 32.03 | Up |
| Right Cerebrum | Indoleacetic acid | -10.89 | Down |
|  | Inosine | -53.75 | Down |
|  | Methylamine | -5.68 | Down |
|  | Methylmalonic acid | -73.72 | Down |
|  | N-Acetylornithine | 15.74 | Up |
|  | Nicotinic acid | -36.52 | Down |
|  | Saccharopine | 18.65 | Up |
|  | S-Adenosylhomocysteine | 25.81 | Up |
|  | L-Tryptophan | 54.09 | Up |
|  | Tyramine | 47.71 | Up |
|  | Xanthurenic acid | -10.39 | Down |
|  | 1-Methylhistidine | -60.98 | Down |
