# Supplementary material for: Early life stress shifts critical periods and causes precocious visual cortex development
Source: PLoS One. 2024 Dec 31;19(12):e0316384. doi: 10.1371/journal.pone.0316384 (PMC11687811; doi:10.1371/journal.pone.0316384)
Supplement: S4 Table — ‡ Indicates pathways altered uniquely in either left or right cerebral hemispheres. (DOCX) [file pone.0316384.s004.docx]

**Supplemental Table 4.** mRNA-Metabolite Joint Pathway Analysis visualizing significant genes and metabolites enriched in pathways across left and right cerebra of adult animals. ‡Indicates pathways altered uniquely in either left or right cerebral hemispheres.

| **Left Cerebrum** | | **Right Cerebrum** | |
| --- | --- | --- | --- |
| **Pathway** | ***p*-Value** | **Pathway** | ***p*-Value** |
| ABC transporters | 4.72E-19 | ABC transporters | 9.75E-20 |
| Central carbon metabolism in cancer | 3.67E-18 | Protein digestion and absorption | 2.02E-17 |
| Protein digestion and absorption | 3.00E-16 | Central carbon metabolism in cancer | 2.15E-16 |
| Aminoacyl-tRNA biosynthesis | 3.84E-14 | Aminoacyl-tRNA biosynthesis | 1.13E-12 |
| Mineral absorption | 1.24E-09 | Mineral absorption | 1.07E-08 |
| Alanine, aspartate and glutamate metabolism | 8.77E-09 | Alanine, aspartate and glutamate metabolism | 5.73E-08 |
| Glycine, serine and threonine metabolism | 7.80E-08 | Glycine, serine and threonine metabolism | 4.97E-07 |
| Taurine and hypotaurine metabolism | 2.16E-07 | Taurine and hypotaurine metabolism | 8.19E-07 |
| beta-Alanine metabolism | 2.32E-07 | beta-Alanine metabolism | 1.13E-06 |
| Pantothenate and CoA biosynthesis | 1.20E-06 | Histidine metabolism | 2.10E-06 |
| Glyoxylate and dicarboxylate metabolism | 2.15E-06 | Arginine biosynthesis | 2.83E-06 |
| Nicotinate and nicotinamide metabolism | 2.93E-06 | Sulfur metabolism | 3.58E-06 |
| Valine, leucine and isoleucine biosynthesis | 4.27E-06 | Pantothenate and CoA biosynthesis | 4.48E-06 |
| Arginine biosynthesis | 2.60E-05 | Valine, leucine and isoleucine biosynthesis | 1.23E-05 |
| Sulfur metabolism | 3.13E-05 | Nicotinate and nicotinamide metabolism | 1.38E-05 |
| Cysteine and methionine metabolism | 1.08E-04 | Butanoate metabolism | 3.34E-05 |
| Arginine and proline metabolism | 1.79E-04 | Cysteine and methionine metabolism | 3.43E-05 |
| Butanoate metabolism | 1.85E-04 | Arginine and proline metabolism | 6.27E-05 |
| Pyruvate metabolism | 1.85E-04 | Glyoxylate and dicarboxylate metabolism | 1.40E-04 |
| Histidine metabolism | 2.07E-04 | Purine metabolism‡ | 2.33E-04 |
| Neuroactive ligand-receptor interaction | 2.23E-04 | Neuroactive ligand-receptor interaction | 2.83E-04 |
| Phenylalanine metabolism | 3.77E-04 | Pyruvate metabolism | 5.10E-04 |
| Pyrimidine metabolism | 1.65E-03 | Pyrimidine metabolism | 5.14E-04 |
| D-Glutamine and D-glutamate metabolism | 2.20E-03 | Propanoate metabolism | 9.35E-04 |
| Propanoate metabolism | 4.55E-03 | Phenylalanine metabolism | 1.02E-03 |
| Retrograde endocannabinoid signaling | 4.97E-03 | Phenylalanine, tyrosine and tryptophan biosynthesis | 1.58E-03 |
| Synaptic vesicle cycle | 5.92E-03 | Valine, leucine and isoleucine degradation | 1.90E-03 |
| GABAergic synapse | 7.72E-03 | D-Glutamine and D-glutamate metabolism | 3.69E-03 |
| Valine, leucine and isoleucine degradation | 7.72E-03 | Regulation of lipolysis in adipocytes | 6.32E-03 |
| Glutathione metabolism‡ | 8.85E-03 | cAMP signaling pathway‡ | 9.36E-03 |
| Nitrogen metabolism | 9.69E-03 | Synaptic vesicle cycle | 1.22E-02 |
| Vitamin B6 metabolism‡ | 1.02E-02 | Retrograde endocannabinoid signaling | 1.25E-02 |
| Proximal tubule bicarbonate reclamation | 1.13E-02 | GABAergic synapse | 1.58E-02 |
| Lysine degradation | 1.19E-02 | Nitrogen metabolism | 1.60E-02 |
| Phenylalanine, tyrosine and tryptophan biosynthesis | 1.36E-02 | Morphine addiction‡ | 1.80E-02 |
| Thiamine metabolism‡ | 1.55E-02 | Proximal tubule bicarbonate reclamation | 1.86E-02 |
| Nicotine addiction | 1.62E-02 | Lysine degradation | 2.40E-02 |
| Glucagon signaling pathway | 1.66E-02 | Tyrosine metabolism‡ | 2.57E-02 |
| Citrate cycle (TCA cycle) | 1.96E-02 | Nicotine addiction | 2.64E-02 |
| Cocaine addiction | 2.25E-02 | Taste transduction‡ | 2.74E-02 |
| Glycerophospholipid metabolism‡ | 2.37E-02 | Citrate cycle (TCA cycle) | 3.19E-02 |
| Phosphonate and phosphinate metabolism‡ | 2.80E-02 | Glucagon signaling pathway | 3.29E-02 |
| Primary bile acid biosynthesis‡ | 2.80E-02 | Tryptophan metabolism‡ | 3.29E-02 |
| Regulation of lipolysis in adipocytes | 3.40E-02 | Cocaine addiction | 3.65E-02 |
| Ascorbate and aldarate metabolism‡ | 3.95E-02 | Antifolate resistance‡ | 3.89E-02 |
| Porphyrin and chlorophyll metabolism‡ | 4.00E-02 |  |  |
| Amphetamine addiction‡ | 4.14E-02 |  |  |
| Galactose metabolism‡ | 4.14E-02 |  |  |
