# Supplementary material for: Early life stress shifts critical periods and causes precocious visual cortex development
Source: PLoS One. 2024 Dec 31;19(12):e0316384. doi: 10.1371/journal.pone.0316384 (PMC11687811; doi:10.1371/journal.pone.0316384)
Supplement: S5 Table — Pearson correlations were used to assess the relationship between behaviours indicative of precocious visual behaviour (i.e., less time spent in the deep region of the visual cliff apparatus) and relative concentrations of metabolites found to be significantly altered by stress in a Mann-Whitney U test. Positive correlations indicate that precocious development of depth perception was linked to lower metabolite concentrations, while negative correlations indicate that precocious development of depth perception was linked to higher metabolite concentrations. Metabolites for which more than one NMR resonance peak was identified are represented as metabolite.1, metabolite.2, … metabolite.n. † Indicates metabolites that were significantly correlated to precocious visual behaviour in both left and right cerebra. (DOCX) [file pone.0316384.s005.docx]

**Supplemental Table 5.** Left and right cerebrum metabolites found to be significantly correlated to precocious visual behaviour. Pearson correlations were used to assess the relationship between behaviours indicative of precocious visual behaviour (i.e., less time spent in the deep region of the visual cliff apparatus) and relative concentrations of metabolites found to be significantly altered by stress in a Mann-Whitney U test. Positive correlations indicate that precocious development of depth perception was linked to lower metabolite concentrations, while negative correlations indicate that precocious development of depth perception was linked to higher metabolite concentrations. Metabolites for which more than one NMR resonance peak was identified are represented as metabolite.1, metabolite.2, … metabolite.n. †Indicates metabolites that were significantly correlated to precocious visual behaviour in both left and right cerebra.

| **Region** | **Metabolite** | ***r*** | ***p*** | **Correlation** |
| --- | --- | --- | --- | --- |
| Left Cerebrum | Aspartate.1† | -0.78 | 0.000012 | Negative |
|  | Aspartate.3† | -0.76 | 0.000028 | Negative |
|  | Agmatine.1†, Phenylalanine.1†, Taurine.1† | -0.76 | 0.000029 | Negative |
|  | Agmatine.4† | -0.73 | 0.000070 | Negative |
|  | Aspartate.2† | -0.73 | 0.000074 | Negative |
|  | 2-Aminobutyrate.2, Leucine.2† | -0.72 | 0.000097 | Negative |
|  | Isoleucine.1† | -0.72 | 0.000106 | Negative |
|  | 2-Aminoadipate.1† | -0.71 | 0.000128 | Negative |
|  | Aspartate.5† | -0.71 | 0.000140 | Negative |
|  | Agmatine.2† | -0.71 | 0.000161 | Negative |
|  | Ethanolamine.1†, Homoserine.1†, Uridine.1† | -0.71 | 0.000164 | Negative |
|  | Aspartate.4† | -0.70 | 0.000232 | Negative |
|  | Aspartate.6†, Uridine.4† | -0.69 | 0.000253 | Negative |
|  | Hypoxanthine† | -0.69 | 0.000272 | Negative |
|  | Niacinamide.2† | -0.69 | 0.000293 | Negative |
|  | Glutaric Acid Monomethyl Ester.1 | -0.69 | 0.000309 | Negative |
| Left Cerebrum | Isoleucine.3† | -0.68 | 0.000346 | Negative |
|  | Histidine.10† | -0.67 | 0.000411 | Negative |
|  | Histidine.7† | -0.67 | 0.000455 | Negative |
|  | 5,6-Dihydrouracil.2† | -0.67 | 0.000516 | Negative |
|  | Uridine.5† | -0.66 | 0.000689 | Negative |
|  | Aspartate.8† | -0.65 | 0.000699 | Negative |
|  | Histidine.11† | -0.65 | 0.000707 | Negative |
|  | Histidine.8† | -0.65 | 0.000763 | Negative |
|  | Taurine.2† | -0.65 | 0.000775 | Negative |
|  | 5,6-Dihydrouracil.1† | -0.65 | 0.000793 | Negative |
|  | Histidine.4† | -0.65 | 0.000819 | Negative |
|  | β-Alanine.3† | -0.64 | 0.001050 | Negative |
|  | Uracil.6† | -0.63 | 0.001148 | Negative |
|  | 5,6-Dihydrouracil.3† | -0.63 | 0.001150 | Negative |
|  | Valine.1† | -0.63 | 0.001301 | Negative |
|  | Histidine.6† | -0.63 | 0.001305 | Negative |
|  | Histidine.5† | -0.62 | 0.001648 | Negative |
|  | Valine.4† | -0.62 | 0.001675 | Negative |
|  | Isoleucine.2†, Valine.2† | -0.61 | 0.002013 | Negative |
|  | Phenylalanine.7† | -0.61 | 0.002112 | Negative |
|  | Uracil.7† | -0.61 | 0.002189 | Negative |
|  | Alanine.1†, Homoserine.3† | -0.60 | 0.002271 | Negative |
|  | Nicotinurate.1† | -0.60 | 0.002277 | Negative |
|  | Taurine.3† | -0.60 | 0.002422 | Negative |
|  | Choline.5† | -0.59 | 0.002747 | Negative |
|  | Choline.10† | -0.59 | 0.002902 | Negative |
|  | Valine.3† | -0.59 | 0.002913 | Negative |
|  | Phenylalanine.8† | -0.59 | 0.003008 | Negative |
|  | 2-Oxoglutarate.1† | -0.59 | 0.003244 | Negative |
|  | 2-Aminoadipate.2† | -0.59 | 0.003333 | Negative |
|  | Homoserine.17†, Uridine.7† | -0.58 | 0.003576 | Negative |
|  | 2-Oxoglutarate.2† | -0.58 | 0.003611 | Negative |
|  | Pyridoxine | -0.58 | 0.003686 | Negative |
|  | 2-Oxoglutarate.3† | -0.58 | 0.003901 | Negative |
|  | 2-Aminobutyrate.5, Leucine.6† | -0.58 | 0.003928 | Negative |
|  | Histidine.1† | -0.57 | 0.004646 | Negative |
|  | β-Alanine.2† | -0.57 | 0.004684 | Negative |
| Left Cerebrum | Aspartate.9†, Uridine.8† | -0.56 | 0.005907 | Negative |
|  | 2-Aminoadipate.3† | -0.56 | 0.005935 | Negative |
|  | Alanine.6†, Homoserine.15† | -0.55 | 0.006075 | Negative |
|  | Homoserine.2† | -0.55 | 0.006559 | Negative |
|  | Phenylalanine.4† | -0.55 | 0.006869 | Negative |
|  | Choline.3† | -0.54 | 0.007268 | Negative |
|  | Phenylalanine.5† | -0.54 | 0.007283 | Negative |
|  | Homoserine.5† | -0.54 | 0.007480 | Negative |
|  | β-Alanine.1† | -0.54 | 0.007668 | Negative |
|  | Uracil.5† | -0.53 | 0.009576 | Negative |
|  | Phenylalanine.3† | -0.53 | 0.009701 | Negative |
|  | Phenylalanine.6† | -0.53 | 0.009892 | Negative |
|  | Histidine.2† | -0.52 | 0.011065 | Negative |
|  | Ethanolamine.4† | -0.51 | 0.012025 | Negative |
|  | Choline.1† | -0.51 | 0.012210 | Negative |
|  | Uracil.4† | -0.51 | 0.012632 | Negative |
|  | Alanine.5†, Homoserine.14† | -0.51 | 0.012669 | Negative |
|  | Phenylalanine.9† | -0.51 | 0.013432 | Negative |
|  | β-Alanine.4† | -0.51 | 0.013762 | Negative |
|  | Choline.2† | -0.51 | 0.013855 | Negative |
|  | 2-Aminobutyrate.1, Leucine.1† | -0.50 | 0.014723 | Negative |
|  | 3-Phenylpropionate.1† | -0.50 | 0.014819 | Negative |
|  | Histidine.9† | -0.50 | 0.015464 | Negative |
|  | β-Alanine.5† | -0.49 | 0.016366 | Negative |
|  | Homoserine.7† | -0.48 | 0.019590 | Negative |
|  | Histidine.3† | -0.48 | 0.019955 | Negative |
|  | Choline.9† | -0.48 | 0.020157 | Negative |
|  | Alanine.3†, Homoserine.11† | -0.48 | 0.021168 | Negative |
|  | β-Alanine.6† | -0.48 | 0.021180 | Negative |
|  | Isoleucine.10† | -0.48 | 0.021963 | Negative |
|  | Homoserine.9† | -0.46 | 0.025610 | Negative |
|  | Choline.4† | -0.46 | 0.026183 | Negative |
|  | Phenylalanine.12† | -0.46 | 0.027698 | Negative |
|  | Agmatine.3†, Leucine.3† | -0.46 | 0.028332 | Negative |
|  | Phenylalanine.11† | -0.45 | 0.032205 | Negative |
| Left Cerebrum | Isoleucine.7† | -0.45 | 0.033228 | Negative |
|  | Ethanolamine.3†, Homoserine.6† | -0.44 | 0.033761 | Negative |
|  | Homoserine.8† | -0.44 | 0.036145 | Negative |
|  | 2-Aminoadipate.4†, 4-Aminobutyrate.1, Glutaric Acid Monomethyl Ester.2 | -0.43 | 0.042619 | Negative |
|  | Nicotinurate.3† | -0.42 | 0.044673 | Negative |
|  | Ethanolamine.5†, Phenylalanine.16† | -0.42 | 0.045083 | Negative |
|  | Phenylalanine.13† | -0.42 | 0.047338 | Negative |
|  | Adenosine.20† | 0.42 | 0.047400 | Positive |
|  | Lactate.5† | 0.42 | 0.047989 | Positive |
|  | N-Acetylaspartate.11† | 0.43 | 0.039226 | Positive |
|  | Glutamate.17†, Pyroglutamate.12† | 0.43 | 0.040708 | Positive |
|  | Glycerol.6†, Glycine | 0.43 | 0.041132 | Positive |
|  | Serine.4† | 0.43 | 0.041629 | Positive |
|  | 4-Pyridoxate.1† | 0.44 | 0.033803 | Positive |
|  | Serine.2† | 0.44 | 0.037181 | Positive |
|  | D-Threitol.4†, Myo-Inositol.9† | 0.46 | 0.025697 | Positive |
|  | N-Acetylaspartate.8† | 0.46 | 0.025986 | Positive |
|  | N-Acetylaspartate.9† | 0.46 | 0.027884 | Positive |
|  | Glycerol.5†, Myo-Inositol.4† | 0.48 | 0.020250 | Positive |
|  | Carnitine.5†, Levulinate.3†, Pyroglutamate.14† | 0.48 | 0.021803 | Positive |
|  | Lactate.4†, Threonine.3† | 0.49 | 0.016643 | Positive |
|  | Glutamate.18† | 0.50 | 0.014369 | Positive |
|  | Lactate.3† | 0.50 | 0.015064 | Positive |
|  | Serine.3† | 0.50 | 0.015336 | Positive |
|  | Serine.1† | 0.50 | 0.015567 | Positive |
|  | Glutamate.11† | 0.50 | 0.015813 | Positive |
|  | Myo-Inositol.2† | 0.51 | 0.012210 | Positive |
|  | D-Threitol.2†, Glycerol.4†, Myo-Inositol.3† | 0.51 | 0.013441 | Positive |
|  | D-Threitol.1† | 0.51 | 0.013800 | Positive |
| Left Cerebrum | Glutamate.4†, N-Acetylaspartate.4†, Pyroglutamate.1† | 0.52 | 0.010548 | Positive |
|  | Adenosine.11† | 0.53 | 0.009670 | Positive |
|  | N-Acetylaspartate.2† | 0.53 | 0.009674 | Positive |
|  | Glycerol.3† | 0.54 | 0.008285 | Positive |
|  | Adenosine.17† | 0.55 | 0.006974 | Positive |
|  | Adenosine.18† | 0.56 | 0.005228 | Positive |
|  | N-Acetylaspartate.1† | 0.56 | 0.005342 | Positive |
|  | Adenosine.8† | 0.56 | 0.005578 | Positive |
|  | Adenosine.12† | 0.56 | 0.005748 | Positive |
|  | Adenosine.10† | 0.56 | 0.005801 | Positive |
|  | Carnitine.4†, Levulinate.2†, Pyroglutamate.11† | 0.56 | 0.005842 | Positive |
|  | N-Acetylaspartate.3† | 0.57 | 0.004124 | Positive |
|  | Adenosine.7† | 0.57 | 0.004610 | Positive |
|  | N-Acetylaspartate.5† | 0.57 | 0.004824 | Positive |
|  | O-Phosphocholine.4 | 0.58 | 0.003444 | Positive |
|  | Adenosine.19† | 0.58 | 0.003533 | Positive |
|  | Lactate.2† | 0.59 | 0.002808 | Positive |
|  | Creatinine.2† | 0.59 | 0.002814 | Positive |
|  | Carnitine.1† | 0.59 | 0.002898 | Positive |
|  | O-Phosphocholine.5 | 0.59 | 0.003024 | Positive |
|  | N-Acetylaspartate.7† | 0.59 | 0.003275 | Positive |
|  | Adenosine.15† | 0.59 | 0.003330 | Positive |
|  | Adenosine.16† | 0.60 | 0.002423 | Positive |
|  | Adenosine.14† | 0.61 | 0.001812 | Positive |
|  | Formate† | 0.63 | 0.001137 | Positive |
|  | Lactate.1† | 0.63 | 0.001271 | Positive |
|  | N-Acetylaspartate.14† | 0.64 | 0.000927 | Positive |
|  | Adenosine.6† | 0.64 | 0.000955 | Positive |
|  | Tyrosine.3† | 0.64 | 0.001029 | Positive |
|  | Adenosine.3† | 0.66 | 0.000581 | Positive |
|  | Glutamate.9†, Glycerol.2† | 0.67 | 0.000467 | Positive |
|  | Glutamate.6†, Glycerol.1† | 0.67 | 0.000469 | Positive |
|  | Adenosine.2† | 0.67 | 0.000473 | Positive |
|  | Creatine†, Tyrosine.1† | 0.67 | 0.000476 | Positive |
|  | Fumarate† | 0.67 | 0.000479 | Positive |
| Left Cerebrum | Adenosine.5† | 0.67 | 0.000524 | Positive |
|  | Glutamate.13† | 0.68 | 0.000360 | Positive |
|  | N-Acetylaspartate.15† | 0.68 | 0.000387 | Positive |
|  | Adenosine.13† | 0.69 | 0.000256 | Positive |
|  | Glutamate.5† | 0.69 | 0.000256 | Positive |
|  | Glutamate.3† | 0.69 | 0.000285 | Positive |
|  | Glutamate.2† | 0.69 | 0.000285 | Positive |
|  | Glutamate.1† | 0.69 | 0.000286 | Positive |
|  | Glutamate.8† | 0.70 | 0.000179 | Positive |
|  | Adenosine.4† | 0.70 | 0.000182 | Positive |
|  | Adenosine.1† | 0.70 | 0.000203 | Positive |
|  | Pyroglutamate.6† | 0.70 | 0.000215 | Positive |
|  | Adenosine.9† | 0.70 | 0.000226 | Positive |
|  | Glutamate.14† | 0.71 | 0.000158 | Positive |
|  | Glutamate.15† | 0.71 | 0.000168 | Positive |
|  | Glutamate.7† | 0.73 | 0.000067 | Positive |
|  | Tyrosine.2† | 0.73 | 0.000077 | Positive |
|  | Glutamate.10† | 0.74 | 0.000058 | Positive |
|  | Myo-Inositol.1† | 0.75 | 0.000033 | Positive |
|  | Glutamate.12† | 0.75 | 0.000043 | Positive |
| Right | Aspartate.9† | -0.80 | 0.000005 | Negative |
| Cerebrum | Aspartate.5†, Tyramine.5 | -0.80 | 0.000006 | Negative |
|  | Aspartate.4† | -0.79 | 0.000006 | Negative |
|  | Uridine.3† | -0.79 | 0.000007 | Negative |
|  | Aspartate.1† | -0.79 | 0.000007 | Negative |
|  | Aspartate.6†, Uridine.1† | -0.79 | 0.000007 | Negative |
|  | Aspartate.3† | -0.79 | 0.000007 | Negative |
|  | Aspartate.7† | -0.79 | 0.000008 | Negative |
|  | Aspartate.2†, Tyramine.3 | -0.78 | 0.000010 | Negative |
|  | Tyramine.6 | -0.77 | 0.000015 | Negative |
|  | β-Alanine.1† | -0.76 | 0.000027 | Negative |
|  | Aspartate.8†, Uridine.5† | -0.76 | 0.000028 | Negative |
|  | Homoserine.13† | -0.76 | 0.000029 | Negative |
|  | Histidine.6† | -0.75 | 0.000033 | Negative |
|  | Histidine.8† | -0.75 | 0.000039 | Negative |
|  | Histidine.4† | -0.73 | 0.000068 | Negative |
|  | Hypoxanthine† | -0.73 | 0.000069 | Negative |
| Right | Niacinamide.1† | -0.73 | 0.000071 | Negative |
| Cerebrum | Choline.1† | -0.72 | 0.000097 | Negative |
|  | Taurine.1† | -0.72 | 0.000102 | Negative |
|  | Alanine.4† | -0.72 | 0.000115 | Negative |
|  | Homocysteine.2 | -0.72 | 0.000116 | Negative |
|  | Taurine.2† | -0.72 | 0.000122 | Negative |
|  | Leucine.4† | -0.72 | 0.000124 | Negative |
|  | Ethanolamine.1† | -0.71 | 0.000128 | Negative |
|  | Ethanolamine.2†, Homoserine.4† | -0.71 | 0.000147 | Negative |
|  | Choline.2† | -0.71 | 0.000150 | Negative |
|  | Nicotinurate.1† | -0.71 | 0.000166 | Negative |
|  | Histidine.5† | -0.71 | 0.000167 | Negative |
|  | β-Alanine.3† | -0.71 | 0.000170 | Negative |
|  | Phenylalanine.6† | -0.70 | 0.000175 | Negative |
|  | Phenylalanine.1† | -0.70 | 0.000181 | Negative |
|  | Tyramine.4 | -0.70 | 0.000184 | Negative |
|  | Homoserine.2† | -0.70 | 0.000185 | Negative |
|  | N-Acetylornithine.1 | -0.70 | 0.000193 | Negative |
|  | Histidine.3† | -0.70 | 0.000204 | Negative |
|  | 5,6-Dihydrouracil.4† | -0.70 | 0.000215 | Negative |
|  | Uracil.4† | -0.70 | 0.000221 | Negative |
|  | 2-Aminoadipate.4†, Leucine.3† | -0.70 | 0.000222 | Negative |
|  | Phenylalanine.5† | -0.69 | 0.000236 | Negative |
|  | 5,6-Dihydrouracil.5† | -0.69 | 0.000239 | Negative |
|  | Histidine.1† | -0.69 | 0.000248 | Negative |
|  | Homocysteine.1 | -0.69 | 0.000250 | Negative |
|  | 4-Aminobutyrate.1, Acetate.1 | -0.69 | 0.000253 | Negative |
|  | Homocysteine.8 | -0.69 | 0.000261 | Negative |
|  | 2-Aminoadipate.6† | -0.69 | 0.000267 | Negative |
|  | 3-Phenylpropionate.2†, Phenylalanine.7† | -0.69 | 0.000268 | Negative |
|  | Valine.1† | -0.69 | 0.000269 | Negative |
|  | Phenylalanine.3† | -0.69 | 0.000272 | Negative |
|  | 4-Aminobutyrate.2 | -0.69 | 0.000277 | Negative |
|  | Valine.3† | -0.69 | 0.000278 | Negative |
|  | Histidine.2† | -0.69 | 0.000281 | Negative |
|  | Ethanolamine.3† | -0.69 | 0.000283 | Negative |
|  | β-Alanine.2† | -0.69 | 0.000288 | Negative |
| Right | 4-Aminobutyrate.6 | -0.69 | 0.000293 | Negative |
| Cerebrum | Agmatine.2† | -0.69 | 0.000299 | Negative |
|  | β-Alanine.4† | -0.69 | 0.000301 | Negative |
|  | 2-Aminoadipate.3† | -0.69 | 0.000305 | Negative |
|  | 4-Aminobutyrate.7, Agmatine.4† | -0.69 | 0.000306 | Negative |
|  | Taurine.3† | -0.69 | 0.000310 | Negative |
|  | Phenylalanine.2† | -0.68 | 0.000312 | Negative |
|  | 4-Aminobutyrate.3 | -0.68 | 0.000321 | Negative |
|  | 5,6-Dihydrouracil.6† | -0.68 | 0.000325 | Negative |
|  | 4-Aminobutyrate.5 | -0.68 | 0.000328 | Negative |
|  | Tryptophan.2 | -0.68 | 0.000335 | Negative |
|  | Choline.8† | -0.68 | 0.000338 | Negative |
|  | Alanine.2† | -0.68 | 0.000344 | Negative |
|  | Valine.2† | -0.68 | 0.000349 | Negative |
|  | Alanine.1†, Isoleucine.4† | -0.68 | 0.000355 | Negative |
|  | Uracil.1† | -0.68 | 0.000371 | Negative |
|  | 5,6-Dihydrouracil.2† | -0.68 | 0.000372 | Negative |
|  | Agmatine.5† | -0.68 | 0.000379 | Negative |
|  | Leucine.1† | -0.68 | 0.000384 | Negative |
|  | Valine.4† | -0.68 | 0.000394 | Negative |
|  | Ethanolamine.6†, Homoserine.10†, Uridine.7† | -0.68 | 0.000398 | Negative |
|  | Tyramine.1 | -0.68 | 0.000410 | Negative |
|  | 3-Phenylpropionate.3†, Phenylalanine.8† | -0.67 | 0.000438 | Negative |
|  | 3-Phenylpropionate.1†, Phenylalanine.4† | -0.67 | 0.000448 | Negative |
|  | Tryptophan.1 | -0.67 | 0.000494 | Negative |
|  | 2-Oxoglutarate.1†, 4-Aminobutyrate.9 | -0.67 | 0.000502 | Negative |
|  | Tyramine.2 | -0.67 | 0.000506 | Negative |
|  | Alanine.3†, Homoserine.5† | -0.67 | 0.000524 | Negative |
|  | β-Alanine.5† | -0.67 | 0.000528 | Negative |
|  | Taurine.5† | -0.66 | 0.000559 | Negative |
|  | Histidine.9† | -0.66 | 0.000560 | Negative |
|  | Saccharopine.1 | -0.66 | 0.000561 | Negative |
|  | 4-Aminobutyrate.8 | -0.66 | 0.000571 | Negative |
|  | Uracil.2† | -0.66 | 0.000598 | Negative |
| Right | Homocysteine.3 | -0.66 | 0.000650 | Negative |
| Cerebrum | Homoserine.6† | -0.65 | 0.000698 | Negative |
|  | Agmatine.1†, Leucine.2† | -0.65 | 0.000730 | Negative |
|  | Homoserine.8† | -0.65 | 0.000745 | Negative |
|  | Homocysteine.5, Isoleucine.6† | -0.65 | 0.000759 | Negative |
|  | Uridine.6† | -0.65 | 0.000784 | Negative |
|  | Isoleucine.1† | -0.65 | 0.000789 | Negative |
|  | Uracil.3† | -0.65 | 0.000806 | Negative |
|  | 5,6-Dihydrouracil.1† | -0.65 | 0.000822 | Negative |
|  | S-Adenosylhomocysteine.1 | -0.65 | 0.000828 | Negative |
|  | 2'-Deoxyadenosine | -0.64 | 0.000929 | Negative |
|  | Homocysteine.4, Isoleucine.5† | -0.64 | 0.000932 | Negative |
|  | Saccharopine.3 | -0.64 | 0.000940 | Negative |
|  | Homoserine.1†, Isoleucine.7† | -0.64 | 0.000990 | Negative |
|  | Isoleucine.3† | -0.64 | 0.001003 | Negative |
|  | Histidine.7† | -0.64 | 0.001016 | Negative |
|  | 2-Aminoadipate.1† | -0.64 | 0.001019 | Negative |
|  | Isoleucine.2† | -0.64 | 0.001112 | Negative |
|  | Choline.4† | -0.63 | 0.001214 | Negative |
|  | Agmatine.3† | -0.63 | 0.001229 | Negative |
|  | 4-Aminobutyrate.4 | -0.63 | 0.001271 | Negative |
|  | Saccharopine.2 | -0.63 | 0.001306 | Negative |
|  | 5,6-Dihydrouracil.3† | -0.63 | 0.001315 | Negative |
|  | Homocysteine.6 | -0.63 | 0.001327 | Negative |
|  | Choline.6† | -0.63 | 0.001384 | Negative |
|  | Choline.7† | -0.63 | 0.001428 | Negative |
|  | Uridine.2† | -0.62 | 0.001458 | Negative |
|  | 2-Aminoadipate.2† | -0.62 | 0.001569 | Negative |
|  | Choline.3† | -0.62 | 0.001698 | Negative |
|  | Homoserine.9† | -0.61 | 0.001969 | Negative |
|  | Isoleucine.9† | -0.61 | 0.002037 | Negative |
|  | Homoserine.3†, Isoleucine.10† | -0.61 | 0.002061 | Negative |
|  | Isoleucine.13† | -0.61 | 0.002148 | Negative |
| Right Cerebrum | S-Adenosylhomocysteine.2 | -0.60 | 0.002283 | Negative |
|  | Agmatine.7†, Phenylalanine.10† | -0.60 | 0.002488 | Negative |
|  | Alanine.5† | -0.60 | 0.002700 | Negative |
|  | Homoserine.11† | -0.59 | 0.002871 | Negative |
|  | Ethanolamine.5† | -0.59 | 0.003036 | Negative |
|  | Tryptophan.3 | -0.59 | 0.003244 | Negative |
|  | Niacinamide.2† | -0.59 | 0.003252 | Negative |
|  | 2-Aminoadipate.5† | -0.58 | 0.003631 | Negative |
|  | Homocysteine.7 | -0.57 | 0.004142 | Negative |
|  | N-Acetylornithine.2 | -0.57 | 0.004177 | Negative |
|  | Isoleucine.8† | -0.57 | 0.004275 | Negative |
|  | Choline.5† | -0.57 | 0.004485 | Negative |
|  | S-Adenosylhomocysteine.3 | -0.57 | 0.004599 | Negative |
|  | Ethanolamine.4†, Homoserine.7†, Uridine.4† | -0.55 | 0.006217 | Negative |
|  | Uridine.8† | -0.55 | 0.006270 | Negative |
|  | S-Adenosylhomocysteine.4 | -0.55 | 0.006840 | Negative |
|  | Isoleucine.11† | -0.55 | 0.006877 | Negative |
|  | 4-Aminobutyrate.10 | -0.54 | 0.007208 | Negative |
|  | N-Acetylornithine.3 | -0.54 | 0.008170 | Negative |
|  | 3-Phenylpropionate.5† | -0.52 | 0.011520 | Negative |
|  | Homocysteine.9, Isoleucine.12† | -0.51 | 0.012005 | Negative |
|  | 3-Phenylpropionate.6† | -0.49 | 0.016788 | Negative |
|  | Taurine.4† | -0.49 | 0.018520 | Negative |
|  | Isoleucine.16† | -0.48 | 0.019431 | Negative |
|  | β-Alanine.6† | -0.48 | 0.019568 | Negative |
|  | N-Acetylornithine.4 | -0.48 | 0.019621 | Negative |
|  | Tryptophan.4 | -0.47 | 0.022120 | Negative |
|  | Homoserine.15† | -0.47 | 0.022777 | Negative |
|  | Niacinamide.3† | -0.46 | 0.026227 | Negative |
|  | Agmatine.6† | -0.46 | 0.027441 | Negative |
|  | Homoserine.12† | -0.46 | 0.028687 | Negative |
| Right Cerebrum | Aspartate.10†, Homocysteine.11 | -0.45 | 0.033013 | Negative |
|  | N-Acetylornithine.5 | -0.45 | 0.033099 | Negative |
|  | Isoleucine.14† | -0.44 | 0.035009 | Negative |
|  | 3-Phenylpropionate.4†, Phenylalanine.9† | -0.43 | 0.039236 | Negative |
|  | Aspartate.11† | -0.42 | 0.044139 | Negative |
|  | Histidine.10† | -0.42 | 0.044478 | Negative |
|  | Adenine.2, Inosine.11 | 0.41 | 0.049318 | Positive |
|  | N-Acetylaspartate.11†, Pyroglutamate.5† | 0.42 | 0.048339 | Positive |
|  | Serine.2† | 0.43 | 0.038908 | Positive |
|  | Adenosine.13†, Serine.3† | 0.43 | 0.042223 | Positive |
|  | Glycerol.9† | 0.44 | 0.034055 | Positive |
|  | Glycerol.8† | 0.44 | 0.037385 | Positive |
|  | 4-Pyridoxate† | 0.45 | 0.032337 | Positive |
|  | Pyroglutamate.10† | 0.46 | 0.025517 | Positive |
|  | N-Acetylaspartate.12†, Pyroglutamate.6† | 0.46 | 0.027594 | Positive |
|  | Indole-3-Acetate.1 | 0.49 | 0.016517 | Positive |
|  | D-Threitol.8†, Myo-Inositol.6† | 0.49 | 0.016804 | Positive |
|  | Carnitine.7†, Levulinate.5†, Pyroglutamate.11† | 0.50 | 0.014242 | Positive |
|  | Carnitine.5† | 0.50 | 0.014458 | Positive |
|  | Inosine.9, N-Acetylaspartate.15† | 0.50 | 0.016323 | Positive |
|  | Pyroglutamate.3† | 0.51 | 0.012120 | Positive |
|  | Guanosine.6 | 0.51 | 0.012729 | Positive |
|  | Doublet 3.865 ppm | 0.53 | 0.008806 | Positive |
|  | Xanthurenate.2 | 0.53 | 0.008860 | Positive |
|  | Lactate.4† | 0.54 | 0.007864 | Positive |
|  | Glycerol.5†, Myo-Inositol.4† | 0.54 | 0.007948 | Positive |
|  | N-Acetylaspartate.10†, Pyroglutamate.2† | 0.54 | 0.008146 | Positive |
|  | Indole-3-Acetate.2 | 0.55 | 0.006276 | Positive |
|  | Glycerol.6† | 0.55 | 0.006319 | Positive |
|  | D-Threitol.6†, Glycerol.7†, Myo-Inositol.5† | 0.56 | 0.005477 | Positive |
| Right  Cerebrum | Lactate.3†, Threonine.1† | 0.56 | 0.005710 | Positive |
|  | D-Threitol.7† | 0.58 | 0.003615 | Positive |
|  | Glutamate.16† | 0.58 | 0.003716 | Positive |
|  | Nicotinate.6 | 0.58 | 0.003754 | Positive |
|  | Pyroglutamate.9† | 0.58 | 0.004102 | Positive |
|  | Xanthurenate.1 | 0.59 | 0.002807 | Positive |
|  | Levulinate.4† | 0.59 | 0.002983 | Positive |
|  | Glutamate.11†, Glycerol.3† | 0.59 | 0.003092 | Positive |
|  | Nicotinate.5 | 0.60 | 0.002233 | Positive |
|  | Guanosine.5 | 0.60 | 0.002739 | Positive |
|  | Carnitine.4† | 0.61 | 0.002019 | Positive |
|  | D-Threitol.9† | 0.61 | 0.002142 | Positive |
|  | Carnitine.2†, Levulinate.2†, Pyroglutamate.7† | 0.62 | 0.001468 | Positive |
|  | Nicotinate.4 | 0.62 | 0.001781 | Positive |
|  | Glutamate.14† | 0.63 | 0.001137 | Positive |
|  | 3-Hydroxybutyrate.2 | 0.63 | 0.001208 | Positive |
|  | 1,3-Dimethylurate.3, Myo-Inositol.3† | 0.63 | 0.001262 | Positive |
|  | Pyroglutamate.8† | 0.63 | 0.001277 | Positive |
|  | Adenosine.7† | 0.63 | 0.001280 | Positive |
|  | Serine.1† | 0.63 | 0.001327 | Positive |
|  | 1,3-Dimethylurate.2 | 0.64 | 0.000979 | Positive |
|  | Lactate.2† | 0.64 | 0.000987 | Positive |
|  | Carnitine.3† | 0.64 | 0.001053 | Positive |
|  | Glutamate.6† | 0.64 | 0.001093 | Positive |
|  | Glutamate.15† | 0.65 | 0.000723 | Positive |
|  | Myo-Inositol.2† | 0.65 | 0.000855 | Positive |
|  | Guanosine.4 | 0.65 | 0.000884 | Positive |
|  | Glutamate.13† | 0.66 | 0.000551 | Positive |
|  | D-Threitol.4† | 0.66 | 0.000561 | Positive |
|  | N-Acetylaspartate.13† | 0.66 | 0.000562 | Positive |
|  | Lactate.1† | 0.66 | 0.000589 | Positive |
|  | Glutamate.17† | 0.66 | 0.000616 | Positive |
|  | Glutamate.9† | 0.66 | 0.000624 | Positive |
|  | Nicotinate.3 | 0.66 | 0.000629 | Positive |
|  | Adenosine.11†, Inosine.8 | 0.66 | 0.000635 | Positive |
| Right  Cerebrum | Adenosine.10†, Inosine.7 | 0.66 | 0.000638 | Positive |
|  | Carnitine.1† | 0.67 | 0.000430 | Positive |
|  | Glutamate.7†, Glycerol.2† | 0.67 | 0.000442 | Positive |
|  | Myo-Inositol.1† | 0.67 | 0.000452 | Positive |
|  | Glutamate.1† | 0.67 | 0.000487 | Positive |
|  | Glutamate.2† | 0.67 | 0.000502 | Positive |
|  | Glutamate.8† | 0.67 | 0.000508 | Positive |
|  | Glutamate.3† | 0.67 | 0.000510 | Positive |
|  | Glutamate.5†, Glycerol.1† | 0.67 | 0.000523 | Positive |
|  | Levulinate.6† | 0.67 | 0.000524 | Positive |
|  | Glutamate.18† | 0.68 | 0.000316 | Positive |
|  | Levulinate.1†, Pyroglutamate.4† | 0.68 | 0.000330 | Positive |
|  | D-Threitol.1† | 0.68 | 0.000337 | Positive |
|  | Glutamate.10† | 0.68 | 0.000375 | Positive |
|  | Fumarate† | 0.68 | 0.000399 | Positive |
|  | Histamine.2 | 0.68 | 0.000400 | Positive |
|  | Guanosine.3, Histamine.1 | 0.69 | 0.000240 | Positive |
|  | Glutamate.4†, N-Acetylaspartate.7†, Pyroglutamate.1† | 0.69 | 0.000243 | Positive |
|  | Adenosine.5† | 0.69 | 0.000289 | Positive |
|  | Guanosine.2 | 0.69 | 0.000298 | Positive |
|  | Caprate.4 | 0.69 | 0.000301 | Positive |
|  | Adenosine.6† | 0.70 | 0.000174 | Positive |
|  | 1,3-Dimethylurate.1 | 0.70 | 0.000174 | Positive |
|  | Formate† | 0.70 | 0.000177 | Positive |
|  | 3-Hydroxybutyrate.1 | 0.70 | 0.000191 | Positive |
|  | Adenosine.4† | 0.70 | 0.000191 | Positive |
|  | Inosine.6 | 0.70 | 0.000217 | Positive |
|  | Inosine.5, N-Acetylaspartate.8† | 0.70 | 0.000219 | Positive |
|  | Caprate.2 | 0.70 | 0.000221 | Positive |
|  | Caprate.1 | 0.70 | 0.000230 | Positive |
|  | N-Acetylaspartate.9† | 0.71 | 0.000129 | Positive |
|  | N-Acetylaspartate.3† | 0.71 | 0.000133 | Positive |
|  | N-Acetylaspartate.6† | 0.71 | 0.000141 | Positive |
|  | Glutamate.12† | 0.71 | 0.000153 | Positive |
| Right  Cerebrum | Adenosine.8†, Creatine†, Tyrosine.1† | 0.71 | 0.000153 | Positive |
|  | D-Threitol.2† | 0.71 | 0.000155 | Positive |
|  | Adenosine.9† | 0.71 | 0.000157 | Positive |
|  | Guanosine.1 | 0.71 | 0.000160 | Positive |
|  | Adenosine.3† | 0.71 | 0.000161 | Positive |
|  | N-Acetylaspartate.5† | 0.71 | 0.000166 | Positive |
|  | N-Acetylaspartate.1† | 0.72 | 0.000094 | Positive |
|  | N-Acetylaspartate.4† | 0.72 | 0.000097 | Positive |
|  | D-Threitol.3† | 0.72 | 0.000110 | Positive |
|  | Inosine.1 | 0.72 | 0.000113 | Positive |
|  | Adenosine.2† | 0.72 | 0.000116 | Positive |
|  | Nicotinate.1 | 0.72 | 0.000119 | Positive |
|  | N-Acetylaspartate.2† | 0.72 | 0.000121 | Positive |
|  | Singlet 1.178 ppm | 0.73 | 0.000067 | Positive |
|  | Methylmalonate.1 | 0.73 | 0.000072 | Positive |
|  | Inosine.2 | 0.73 | 0.000081 | Positive |
|  | Adenosine.1†, Inosine.3 | 0.73 | 0.000082 | Positive |
|  | N-Acetylaspartate.16† | 0.73 | 0.000082 | Positive |
|  | Doublet 3.881 ppm | 0.73 | 0.000086 | Positive |
|  | π-Methylhistidine | 0.74 | 0.000057 | Positive |
|  | Adenine.1 | 0.74 | 0.000062 | Positive |
|  | Nicotinate.2 | 0.75 | 0.000038 | Positive |
|  | Creatinine.1† | 0.75 | 0.000038 | Positive |
|  | Adenosine.12†, Tyrosine.2† | 0.75 | 0.000039 | Positive |
|  | D-Threitol.5† | 0.75 | 0.000039 | Positive |
|  | Inosine.4 | 0.75 | 0.000044 | Positive |
|  | Methylmalonate.2 | 0.77 | 0.000018 | Positive |
|  | N-Acetylaspartate.14† | 0.77 | 0.000020 | Positive |
